# Integrative Analysis of the Transcriptome and Metabolome Reveals Genes Involved in Phenylpropanoid and Flavonoid Biosynthesis in the *Trapa bispinosa* Roxb.

**DOI:** 10.3389/fpls.2022.913265

**Published:** 2022-07-07

**Authors:** Dong-Jie Yin, Shi-Jie Ye, Xiao-Yan Sun, Qin-Yi Chen, Ting Min, Hong-Xun Wang, Li-Mei Wang

**Affiliations:** ^1^College of Life Science and Technology, Wuhan Polytechnic University, Wuhan, China; ^2^College of Food Science and Engineering, Wuhan Polytechnic University, Wuhan, China

**Keywords:** *Trapa bispinosa*, transcriptome, metabolome, phenolic compounds, flavonoid biosynthesis

## Abstract

**Background:**

*Trapa bispinosa* Roxb. is grown worldwide as an important aquatic cash crop. Current research on *Trapa bispinosa* primarily focuses on the separation and identification of active ingredients, as well as the inhibitory effect on tumors; however, research on the molecular mechanism of secondary metabolite accumulation is rather limited. Consequently, an integrative analysis of transcriptome and metabolome is required to identify the key metabolic pathways, and key genes, and to explain the molecular mechanism of *Trapa bispinosa*.

**Results:**

The biosynthesis pathways of phenolics in *Trapa bispinosa* were examined through transcriptome and metabolome analyses. Transcriptome analysis yielded 42.76 million clean reads representing 81,417 unigenes with an average length of 1,752 bp. KEGG pathway analysis revealed that 1,623 unigenes, including 88 candidate unigenes related to phenolics biosynthesis, were up-regulated in *Trapa bispinosa* shell (FR) when compared to leaves (LF), root (RT), and stem (ST). The FR vs. LF group had the highest number of specific genes involved in phenylpropanoid, flavonoid, flavone, and flavonol biosynthesis pathways compared to all other comparison groups. In addition, RNA sequencing revealed 18,709 SSRs spanning 14,820 unigenes and 4,387 unigenes encoding transcription factors. Metabolome analysis identified 793 metabolites, including 136 flavonoids and 31 phenylpropane compounds. In the FR group compared to the LF group, there were 202 differentially accumulated metabolites (DAMs). The combined transcriptome and metabolome analyses indicated a significant correlation between 1,050 differentially expressed genes (DEGs) and 62 DAMs. This view proposes a schematic of flavonoid biosynthesis in the FR vs. LF group, providing evidence for the differences in genes and metabolites between FR and LF.

**Conclusion:**

In this study, through *de novo* transcriptome assembly and metabolome analysis, several DEGs and DAMs were identified, which were subsequently used to build flavonoid biosynthesis pathways and a correlation network. The findings pave the way for future research into the molecular mechanisms and functional characterization of *Trapa bispinosa* candidate genes for phenolics biosynthesis.

## Introduction

*Trapa bispinosa* (water chestnut), also known as Trapa L. (Trapaceae) in Myrtales, is a floating herbaceous hydrotherophyte primarily found in tropical and temperate regions (Lim, 2013; Garg et al., 2020). *Trapa bispinosa* is regarded as an important aquatic economic plant in many European, Asian, and North American countries (Kukuła and Bylak, 2017). *Trapa bispinosa* was first cultivated in China around three thousand years ago, and it is today primarily distributed in the middle and lower reaches of the Yangtze River (Garg et al., 2020). It has been demonstrated that the nut of *Trapa bispinosa* is edible and beneficial to the spleen and stomach (Flora of China Editorial Committee, 2018).

The non-edible part of *Trapa bispinosa* can also be used as a traditional medicine for astringent intestinal laxatives, detoxifying heat, esophageal cancer, dysentery, and other common diseases (Khare, 2008). Emerging evidence suggests that alcohol extracts of *Trapa bispinosa* shell (FR) are effective in the treatment of gastric ulcers (Kar et al., 2010), bacteria, infections (Biswas et al., 2012; Razvy et al., 2012), immunoregulation, and neuroprotection (Ambikar et al., 2010), diabetes mellitus, and cataracts (Kinoshita et al., 2019). Further analysis revealed that FR extracts contain a high concentration of active phenolic compounds, including 3-O-methyl gallic acid, chlorogenic acid, caffeic acid, and ferulic acid (Stoicescu et al., 2012). Current research on *Trapa bispinosa* has primarily focused on the characterization of active ingredients and pharmacological action, with little attention paid to the biosynthesis pathway and molecular mechanism of phenolics with various physiological activities.

In plants, phenolic compounds are a class of secondary metabolites that are widely distributed and have a complex structure (Roleira et al., 2018). The majority of phenolic compounds are believed to be derived from the aromatic amino acid phenylalanine metabolism (Swanson, 2003). Numerous plant-derived phenolics exhibit a variety of biological activities, including antioxidant (Rice-Evans et al., 1997; Swallah et al., 2020; Yan Z. et al., 2020), anti-virulence (Bhattacharya et al., 2020), anti-tumor (Jeong et al., 2017; Yoo et al., 2020), anti-obesity (Rodríguez-Pérez et al., 2019), anti-aging (Wojdyło et al., 2018), etc. The first steps in the biosynthesis of plant phenolics are glycolysis and pentose-phosphate pathways (Laura et al., 2019). Initially, phosphoenolpyruvate (PEP) and erythritol-4-phosphate (E4P) produced phenylalanine, tyrosine, and tryptophan *via* the shikimic acid pathway; these aromatic amino acids are then involved in secondary metabolic pathways such as phenylpropane and flavonoids biosynthesis, where they are transformed into various secondary metabolites, thereby playing an important role in numerous physiological activities, including plant growth and development.

High-throughput technologies, such as genome (Meng et al., 2020), transcriptome (Stark et al., 2019), and metabolome (Zampieri et al., 2017; Vignoli et al., 2019), are widely used to extract in-depth information from plants, animals, and microorganisms due to their high efficiency, speed, and accuracy. In recent years, integrative transcriptome and metabolome analysis has played an important role in the regulation of plant growth and development (Xin et al., 2019; Zhang et al., 2020; Wu et al., 2021). In this study, a combination of transcriptome and metabolome analyses demonstrated that FR is the primary tissue involved in the biosynthesis of phenolic compounds. In addition, several genes involved in the biosynthesis of phenolic compounds in FR were identified, and a mechanism for the biosynthesis of flavonoids in FR was proposed. The discovery of related genes and early characterization of flavonoid biosynthesis pathways provide a theoretical basis for future research into the molecular mechanisms of flavonoid biosynthesis pathways in FR.

## Materials and Methods

### Plant Materials

*Trapa bispinosa* served as the experimental material, and samples were collected in Wuhan, China (N30°27′, E114°10′) in 2020. The samples were collected from RT, ST, LF, and FR in September when the nuts were mature and ready for harvest. *Trapa bispinosa*, which has a consistent growth status during this period, was sampled; 12 samples from FR, LF, RT, and ST (three biological repetitions) for transcriptome analysis and 6 samples from FR and LF (three biological repetitions) for metabolome analysis. All samples were prepared, immediately frozen with liquid nitrogen, and stored in a –80°C refrigerator.

### Total RNA Extraction and Transcriptome Analysis

Total RNA was extracted using the Trizol reagent from all samples. The quality of total RNA was determined using agarose gel electrophoresis and a NanoDrop micro-spectrophotometer. Following that, 12 cDNA libraries (FR_1, FR_2, FR_3, LF_1, LF_2, LF_3, ST_1, ST_2, ST_3, RT_1, RT_2, RT_3) were constructed. The DNBSEQ sequencing platform (BGI-Shenzhen, China) was used for RNA-Seq. The RNA sequencing data were deposited in the Sequence Read Archive (SRA) of NCBI (accession number: PRJNA831559). SOAPnuke (v1.4.0) was used to clean the raw reads by eliminating adaptors, low quality, or poly-N reads (Chen et al., 2018). The clean reads were assembled using Trinity (v2.0.6) (Grabherr et al., 2011). To obtain unique genes, Tgicl (v2.0.6) was used to perform clustering and remove redundant data from assembled transcripts (Pertea et al., 2003). The assembled transcripts were then analyzed for expression and functional annotation.

### Function Annotation and Differential Expression Analysis of Transcriptome

Bowtie2 (v2.2.5) (Langmead and Salzberg, 2012) was used for clean-read alignment, with the following parameters: -q –phred64 –sensitive –dpad 0 –gbar 99999999 –mp 1,1 –np 1 –score-min L,0,-0.1 -p 16 -k 200. Under default parameters, the RSEM (v1.2.8) (Li and Dewey, 2011) was used to determine gene expression normalized to FPKM (Fragments per kilobase of transcript per million mapped reads). The functional annotation of unigenes was achieved by mapping assembled genes to several databases, including NT, NR, KOG, and KEGG using the BLAST (v2.2.23) software and an *E*-value threshold < 10^–5^ (Altschul et al., 1990). Blast2GO (v2.5.0) was used to annotate GO with NR annotations. DEGs were identified using DEseq2 (Love et al., 2014) with a broad threshold (|fold change (FC)| > 2 and adjusted *P*-value ≤ 0.01). Phyper, a function of R, was used to perform GO and KEGG enrichment analysis. The *Q*-value with a stringent threshold (*Q*-value < 0.05) was used to adjust the significant levels of terms and pathways. After identifying the CDS section of unigenes with ESTScan and translating them into protein sequences, transcription factors were identified using PlantTFDB.

### Identifying Simple Sequence Repeats

MISA Perl (v1.0) (Thiel et al., 2003) was used to identify simple sequence repeats (SSRs) in all assembly unigenes. The screening statistics consisted of the repeating unit of Mono-nucleotide (Duplicate Times > 12), Di-nucleotide (Duplicate Times > 6), Tri-nucleotide (Duplicate Times > 5), Quad-nucleotide (Duplicate Times > 5), Penta-nucleotide (Duplicate Times > 4) and Hexa-nucleotide (Duplicate Times > 4). A composite microsatellite was formed when the distance between two microsatellites was less than 100 bp.

### Quantitative Real-Time PCR Analysis

To validate the transcriptome data, 31 unigenes were randomly selected for real-time quantitative PCR (qRT-PCR) based on the differences in the expression level of unigenes between each comparison group (FR-vs.-LF, FR-vs.-RT, FR-vs.-ST, LF-vs.-RT, LF-vs.-ST, RT-vs.-ST). Primer Premier 5.0 was used to design the primers of each unigene (Supplementary Table 1), and three technical replicates were performed for each gene from three biological replicate samples. cDNA was synthesized using PrimeScript™ II 1st Strand cDNA Synthesis Kit (TaKaRa, Japan). Primers for each selected unigene were designed using Primer Premier 5.0, and the length of the amplified products ranged from 80 to147 bp. Genomic DNA was extracted using the PrimeScript™ RT reagent Kit with gDNA Eraser (Perfect Real Time) (TaKaRa, Japan), and then subjected to qRT-PCR using TB Green^^®^^ Premix Ex Taq™ II (TIi RNaseH Plus) (TaKaRa, Japan) in Bio-Rad CFX96™ system (Bio-Rad, America). EIF5A (CL1168.Contig2) was generated as an internal reference gene. The relative expression multiples of each gene in different samples were calculated using the 2^–ΔΔCt^ method.

### Sample Preparation and Extraction

The freeze-dried samples were crushed using a mixer mill for 30 s at 45 Hz. 50 mg aliquots of individual samples were weighed and submerged in 700 μL of the extract solution (methanol/water = 3:1). The samples were extracted overnight at 4°C on a shaker following a 30 s vortex. The sample was then centrifuged at 13,000 g for 15 min. The supernatant was carefully filtered through a 0.22 μm microporous membrane, and the resultant supernatants were stored at –80°C for the UHPLC- MS analysis by ShangHai BioTree Biotechnology Co., Ltd., China.

### UHPLC-MS Analysis of Metabolites

The UHPLC separation was performed using the EXIONLC System (Sciex). The mobile phase A consisted of 1% formic acid in water, while the mobile phase B was acetonitrile. The column temperature was set at 40°C. The auto-sampler temperature was set at 4°C and the injection volume was 2 μL. A Sciex QTrap 6500 + (Sciex Technologies) was used for assay development. Ion source parameters were as follows: IonSpray Voltage: +5,500/-4,500 V, Curtain Gas: 35 psi, Temperature: 400^°^C, Ion Source Gas 1:60 psi, Ion Source Gas 2: 60 psi, DP: ± 100 V.

### Identification of Metabolites and Statistical Analyses

The SCIEX Analyst Work Station Software (Version 1.6.3) was used for data collection and processing. MSconverter was used to convert MS raw data (.wiff) to TXT format. Peak detection and annotation were performed using an in-house R program and database (Zhang et al., 2015). SIMCA (V16.0.2) was used to perform principal component analysis (PCA) and orthogonal projections to latent structures-discriminant analysis (OPLS-DA) (Wiklund et al., 2008).

The value of variable importance in the projection (VIP) of the first principal component in OPLS-DA analysis was obtained to visualize group separation and identify significantly changed metabolites. It summarizes the contribution of each variable to the model. Metabolites with VIP > 1 and fold change ≥ 2 or ≤ 0.5 and *P*-value < 0.05 (Student’s *t*-test) were considered DAMs. Commercial databases, including KEGG and MetaboAnalyst,^1^ were used for KEGG enrichment analysis.

### Correlation Analysis of the Transcriptome and Metabolome

Pearson product-moment correlation coefficient tests were used to calculate the correlation coefficient between DEGs and DAMs associated with the flavonoid and phenylpropane biosynthesis pathways in the FR-vs.-LF comparison groups. The Pearson correlation coefficient (PCC) ≥ 0.7 and the *P*-value ≤ 0.5 were chosen and visualized using a nine-quadrant graph. The correlation network was used to identify potential key genes and metabolites, with PCC > 0.9 and |log_2_(FC)| > 2 as screening criteria. Furthermore, the DEGs and DAMs in the FR-vs.-LF groups were mapped to the KEGG database to obtain a common pathway, and the regulatory network of the flavonoid biosynthesis pathway in *Trapa bispinosa* was proposed based on the up and/or downregulation of genes and metabolites.

## Results

### Transcriptome Sequencing and *de novo* Sequence Assembly of *Trapa bispinosa*

Total RNA isolated from the roots (RT), stems (ST), leaves (LF), and shell (FR) of *Trapa bispinosa* was used to construct a cDNA library for sequencing (Supplementary Table 2). Paired-end sequencing with the DNBSEQ platform yielded 76.97 Gb of original data, and 43.82 million original reads were obtained from the four libraries. All samples showed Q20 values greater than 96.59%, and a Q30 value greater than 91.33% (Supplementary Table 3).

After the reads were assembled using Trinity and redundant data eliminated, the FR, LF, RT, and ST yielded 65,587, 65,865, 66,835, and 66,796 transcripts, respectively. After summarizing the unigenes from all samples, redundant data were eliminated using RSEM, and 81,417 unigenes were finally obtained for functional annotation analysis. The N50 of each sample was greater than 1,710 bp, indicating an adequate level of assembly (Supplementary Table 4). The lengths of all unigenes and CDS are shown in Supplementary Figures 1, 2.

### Functional Annotation of Unigenes

The annotation findings for all the unigenes were compared to seven public databases, including NR, NT, KOG, Swiss-Prot, Pfam, GO, and KEGG, as shown in Table 1. The number of annotated unigenes in all databases was 35,895 (44.09%), while the number of annotated unigenes in at least one database was 74,514 (91.25%). In addition, 6,903 unigenes were not annotated to any databases, indicating that they may be novel genes with unknown functions. Among the seven databases, NR had the highest annotation rate. An estimated 73,217 unigenes had been annotated, accounting for 89.93% of all unigenes. According to Engler’s classification, the top three species with annotated rates were *Punica granatum* (82.72%), *Syzygium oleosum* (1.28%), and *Eucalyptus grandis* (1.27%) (Supplementary Figure 3), all belonging to Myrtales. Pomegranate (*Punica granatum* L.) had a high annotation rate, which could be attributed to the completeness of the species’ genomic information in the NR database or the genetic relationship between *Trapa bispinosa* and Pomegranate. The indicators of species distribution in NR database, the *E*-value distribution and similarity distribution in Swiss-Prot database (Supplementary Figure 4) exhibited high-quality annotation and can be used for subsequent analysis.

**TABLE 1:** The details of annotation in different databases about *Trapa bispinosa.*

Items	Number of unigenes	Percentage (%)
Annotated in NR	73,217	89.93
Annotated in NT	61,346	75.35
Annotated in Swiss-Prot	59,539	73.13
Annotated in KEGG	59,956	73.64
Annotated in KOG	60,969	74.88
Annotated in Pfam	59,997	73.69
Annotated in GO	56,131	68.94
Intersection unigenes	35,895	44.09
Annotated in all databases	74,514	91.52
Total unigenes	81,417	100.00

**FIGURE 1:**
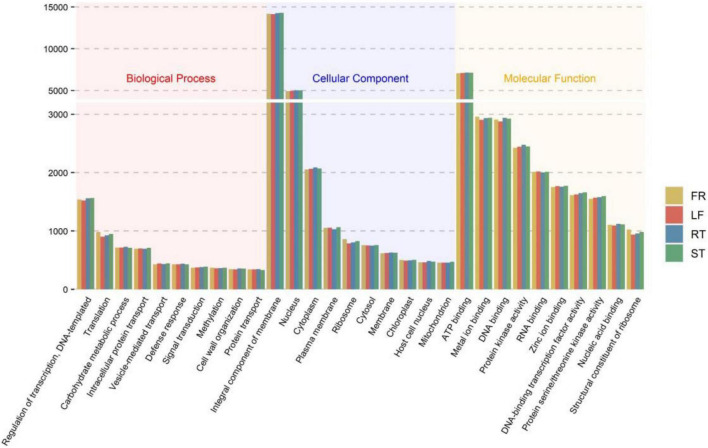
GO classification of *Trapa bispinosa* transcriptome.

In addition, 56,131 (68.94%) unigenes were annotated in the GO database. All unigenes were classified according to their biological processes, cell components, and molecular functions. The results indicated that 26,393 unigenes were annotated to 1,855 different GO terms under biological processes, 33,246 unigenes to 540 different GO terms under cell components, and 44,294 unigenes to 1,619 different GO terms under molecular functions. Figure 1 depicts the top ten GO terms in each category. Supplementary Table 5 displays the top 300 GO entries for each of the four different parts of the *Trapa bispinosa* in each GO category.

The KEGG database was used to classify and annotate the unigenes to further investigate their function. The unigenes were assigned to 139 pathways (Supplementary Table 6), and the top 15 pathways containing the highest number of unigenes in these tissues were Plant hormone signal transduction, RNA transport, and Plant-pathogen pathways (Supplementary Figure 5). Additionally, the KEGG enrichment analysis of the biosynthesis of other secondary metabolites in the KEGG classification of total unigenes revealed that 1,235 unigenes were enriched in 79 metabolic pathways associated with phenolic compounds (Supplementary Figure 6). There were 1,067 unigenes involved in biosynthesis, including Phenylpropanoid biosynthesis (ko00940), Flavonoid biosynthesis (ko00941), Tropane, piperidine, and pyridine alkaloid biosynthesis (ko00960), Flavone and flavonol biosynthesis (ko00944) pathway, Stilbenoid, diarylheptanoid, gingerol biosynthesis (ko00945), and Anthocyanin biosynthesis (ko00942) (Supplementary Table 7). Notably, the expression level of unigenes associated with phenolic compounds biosynthesis exhibit tissue specificity in four different *Trapa bispinosa* tissues, with the expression level of unigenes associated with phenolic compounds biosynthesis in FR being greater than in the other three tissues (Supplementary Figure 7). These findings show that specific genes may exist in different *Trapa bispinosa* tissues and that the FR tissue in *Trapa bispinosa* may be the primary organ for phenolic compound biosynthesis.

### Identification of Simple Sequence Repeats

**FIGURE 2:**
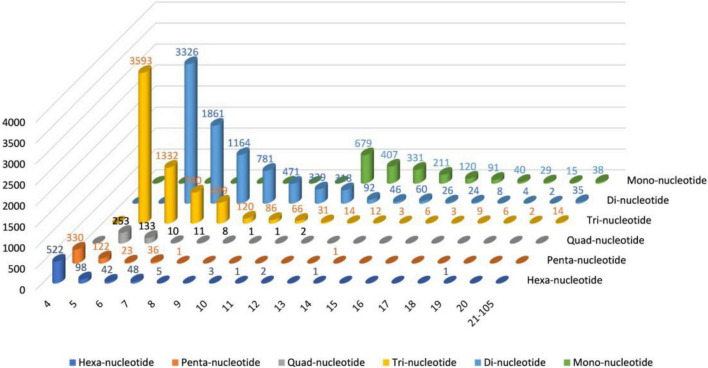
The distribution of SSRs in *Trapa bispinosa*.

The MISA software was used to identify single to six simple sequence repeats from transcriptome data to identify molecular markers of *Trapa bispinosa* for future genome analysis and research; a total of 18,709 SSRs (Supplementary Table 8 and Figure 2) were obtained. The number of two-base and three-base repeats is the largest among these SSRs, accounting for 80.67% (15,093) of all SSRs. The number of Mono-nucleotide was primarily concentrated in 12–16 repeats (1,748, 89.14%), the number of Di-nucleotide was primarily concentrated in 6–12 repeats (8,260, 96.53%), and the number of Tri-nucleotide was concentrated in 5–9 repeats (6,284, 96.14%). The findings suggest that the genome of *Trapa bispinosa* may be highly variable.

### Quantitative Real-Time PCR of Validation RNA Sequencing Data

qRT-PCR was used to determine the expression of 31 unigenes from 6 different comparison groups (FR-vs.-LF, FR-vs.-RT, FR-vs.-ST, LF-vs.-RT, LF-vs.-ST, RT-vs.-ST) to validate the accuracy of the transcriptome data. These unigenes were randomly selected from a pool of high expression unigenes. qRT-PCR and transcriptome data demonstrated that these unigenes exhibited the same pattern (Supplementary Figure 8). The correlation coefficient between qRT-PCR and transcriptome sequencing for the 31 unigenes was 0.9739 (*R*^2^ = 0.9739). The results demonstrated that the transcriptome sequencing data on the differential expression of unigenes in various *Trapa bispinosa* tissues was reliable.

### Analysis of Tissue-Specific Genes

According to PCA analysis and Pearson correlation coefficient analyses, there was a strong correlation and discrimination between biological replicates of different tissues (Supplementary Figures 9, 10). To investigate the DEGs between different tissues, a pairwise comparison (Supplementary Figure 11) of different organs under the conditions of *P*-value < 0.05 and |log_2_(FC)| > 2 was performed. The results demonstrated that the number of DEGs between the FR and LF was enormous (9,707), whereas the variability between the RT and ST was relatively small (2,008). In addition, we investigated the related genes in different tissues by screening the specific genes of each tissue based on the criteria *P*-value < 0.05 and |log_2_(FC)| > 2. The statistical analysis revealed 2,098, 1,546, 472, and 122 tissue-specific unigenes (Supplementary Figure 12) in FR, LF, RT, and ST, respectively. Tissue-specific genes in FR accounted for 49.50% of the total genes, including 1,623 up-regulated and 475 down-regulated genes. Next, tissue-specific unigenes were subjected to KEGG enrichment analysis, and significant enrichment pathways in each tissue were identified (Supplementary Table 9). FR was significantly enriched in 21 KEGG pathways, including metabolic pathways for Phenylpropanoid biosynthesis, Flavonoid biosynthesis, Flavone and flavonol biosynthesis. In ST and RT, only three and nine KEGG pathways, respectively, were significantly enriched. In LF, 15 KEGG pathways were significantly enriched, including pathways for Fatty acid elongation, Stilbenoid, diarylheptanoid, and gingerol biosynthesis. These pathways are associated with plant growth in LF. The FR possessed the most specific genes in pathways associated with phenolic biosynthesis, followed by the LF. In the aforementioned statistical data, the number of unigenes associated with phenolic biosynthesis was counted. 88 FR unigenes were found to be up-regulated in the biosynthesis of phenolics, whereas only 8 LF unigenes were found to be up-regulated in the biosynthesis of phenolics. Furthermore, no tissue-specific down-regulation genes associated with FR biosynthesis were identified, but 33 down-regulation genes were identified in LF (Supplementary Figure 13). The data imply that FR and LF are the two tissues with the greatest disparity in phenolics, with FR possibly being the primary site for phenolics biosynthesis.

### Analysis of Differentially Expressed Genes Associated With Phenolics Biosynthesis

**FIGURE 3:**
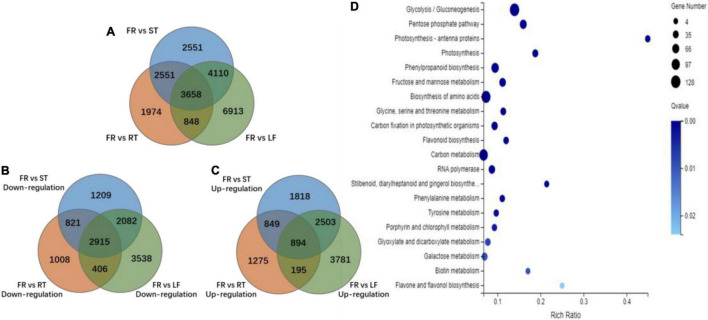
Identification of specific unigenes in FR of *Trapa bispinosa* by DEGs analysis. **(A)** The Venn diagram of identified DEGs in each group. **(B)** The Venn diagram of DEGs that were only up-regulated in FR. **(C)** The Venn diagram of DEGs that were only down-regulated in FR. **(D)** Statistical analysis of KEGG pathway enrichment for DEGs that were only up-regulated in FR.

After taking the intersection of the DEGs in FR-vs.-LF, and FR-vs.-RT, FR-vs.-ST, the number of unigenes with tissue specificity in FR was determined to be 3,658 (Figure 3A). There were 2,915 upregulated and 894 s downregulated unigene (Figures 3B,C) in FR. KEGG enrichment was performed on *Trapa bispinosa* unigenes with an up-regulation tendency (*Q*-value < 0.05). The results demonstrated that the up-regulated genes are primarily concentrated in the pathways related to phenylpropanoid biosynthesis, flavonoid biosynthesis, stilbenoid, diarylheptanoid, gingerol biosynthesis, and flavonol biosynthesis (Figure 3D); the genes involved are listed in Supplementary Table 10.

### Metabolomic Profiling

We described a metabolic analysis in three replicates of two *Trapa bispinosa* tissue samples (FR and LF). All QC samples had the same retention time and peak area in total ion chromatography (TIC) (Supplementary Figure 14), and all QC samples had a Pearson correlation coefficient (PCC) of 0.98 (Supplementary Figure 15), indicating that the method was stable and the data quality was high. In this study, 887 peaks were detected in FR and LF, and 793 metabolites remained after relative standard deviation de-noising.

**FIGURE 4:**
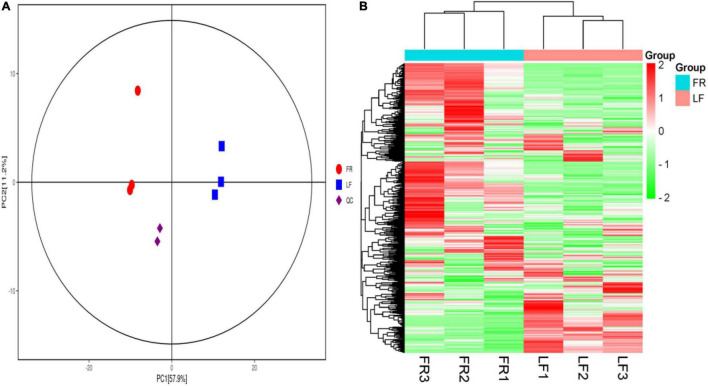
Differential metabolites analysis. **(A)** PCA 3D plot. **(B)** Heatmap based on hierarchical clustering analysis.

All of the metabolites contained a high concentration of phenylpropanoids, lipids, terpene, alkaloids, flavonoids, and lignans (Table 2 and Supplementary Table 11). *Trapa bispinosa* has an active metabolic system, as evidenced by the discovery of a large number of secondary metabolites. Then, using PCA, the overall distribution pattern among all samples was observed. PC1 and PC2 explained 57.9% and 11.2% of the variances between samples, respectively. PC1 and PC2 accounted for 69.1% of the sample differences, suggesting that PCA may cluster different groups of *Trapa bispinosa* samples (Figure 4A). Heatmap clustering results revealed significant differences in metabolite content between FR and LF (Figure 4B). These findings imply that the detection of metabolome was highly reliable.

**TABLE 2:** Categories of the 793 metabolites identified in two tissues of *Trapa bispinosa.*

Category	Number of metabolites
Miscellaneous	146
Flavonoids	136
Terpenoids	91
Alkaloids	76
Phenols	68
Lipids	48
Amino acid and derivatives	42
Phenylpropanoids	31
Steroids and steroid derivatives	30
Coumarins	25
Nucleotide and its derivates	25
Organooxygen compounds	22
Benzene and substituted derivatives	15
Lignans	14
Carboxylic acids and derivatives	12
Xanthones	12

### Identification and Enrichment Analysis of the Differentially Accumulated Metabolites

VIP > 1 and |log_2_(FC)| > 1 were used as the screening criteria of DAMs. The results of the OPLS-DA and 200-response sorting tests (Supplementary Figure 16) indicated that the model was stable and reliable, allowing for a follow-up study to be conducted.

In this study, 202 DAMs from the FR-vs.-LF group were screened. The volcano graph (Supplementary Figure 17) demonstrated that 116 DAMs were up-regulated whereas 86 DAMs were down-regulated (Supplementary Table 12). KEGG enrichment analysis revealed that the DAMs in the FR-vs.-LF group were significantly enriched in the biosynthesis of valine, leucine, and isoleucine, aminoacyl-tRNA biosynthesis, flavonoid biosynthesis, flavone and flavonol biosynthesis, and glutathione metabolism (Supplementary Figure 18).

### Correlation Analysis Between Transcriptome and Metabolome Data

**FIGURE 5:**
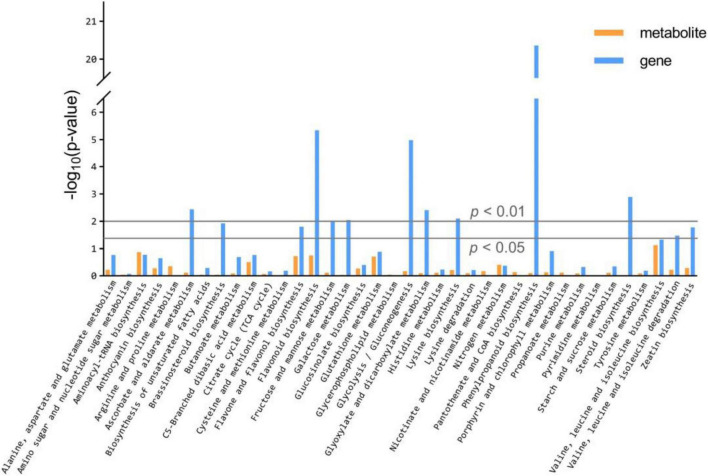
KEGG enrichment analysis of the DEGs and DAMs which were enriched in the same pathway.

The KEGG database enrichment results for DEGs and DAMs show that several DEGs and DAMs are enriched in the same KEGG pathway, including the biosynthesis pathways of valine, leucine, isoleucine, aminoacyl-tRNA, flavonoid, flavone, and flavonol, and the metabolic pathways of glutathione, C5-Branched dibasic acid, nitrogen, arginine, and proline (Figure 5 and Supplementary Table 13).

**FIGURE 6:**
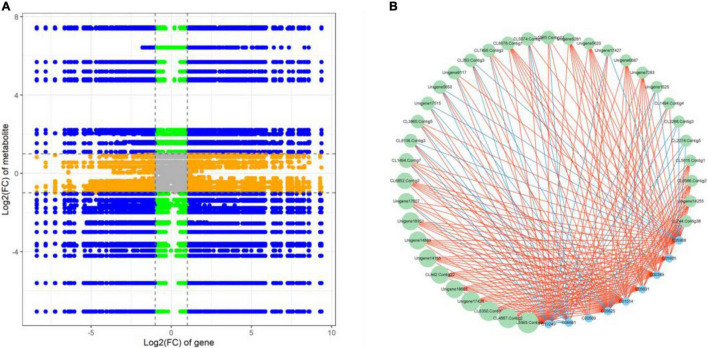
**(A)** A nine-quadrant diagram showing the association of DEGs and DAMs with FR and LF libraries. **(B)** Connection network between DEGs (green circles) and DAMs (blue circles)

Correlation analysis was used to examine the potential regulatory network between DEGs and DAMs. The nine-quadrant graph revealed that several single DAMs were regulated by multiple DEGs, or a single DEG regulated multiple DAMs (Figure 6A). Correlation analysis revealed that 1,050 DEGs were significantly correlated with 62 dams (Supplementary Table 14). L-aspartic acid (C00049) and L-glutamic acid (C00025) were found to be significantly associated with 99 DEGs and 432 DEGs, respectively. The DEG denoted as, CL6477 Contig5 annotated as peroxidase [EC:1.11.1.7], was associated with DAMs such as Luteolin (C01514), Rutin (C05625), Delphinidin (C05908), Eriodictyol (C05631) and Myricetin (C10107). In addition, we discovered that 35 DEGs were significantly correlated with 9 flavonoids associated with DAMs. Notably, Unigene17426, a NAC family gene, was significantly positively correlated (PCC > 0.90) with Quercetin (C00389), Luteolin (C01514), Rutin (C05625), Astragalin (C12249), Delphinidin (C05908), Cyanidin (C05905), and Eriodictyol (C05631), and negatively correlated (PCC < –0.90) with Naringenin chalcone (C06561) (Figure 6B).

**FIGURE 7:**
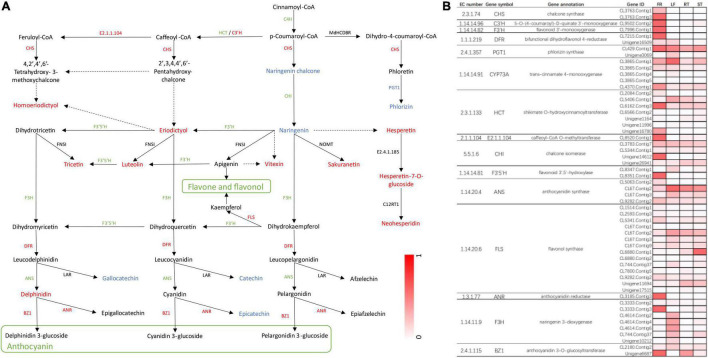
The DEGs and DAMs associated with flavonoid biosynthesis in FR and LF groups of *Trapa bispinosa*. **(A)** Flavonoid biosynthesis pathway. The red letters represent up-regulated DEGs and DAMs. The green letters represent down-regulated DEGs. The blue letters represent down-regulated DAMs. **(B)** Changes in expression of genes involved in flavonoid biosynthesis pathway.

Finally, we constructed flavonoid biosynthesis pathways in FR by referencing flavonoid biosynthesis pathways in the KEGG database with the detected DEGs and DAMs (Figure 7A) and determined gene expression and metabolite accumulation in this pathway between FR and LF. A total of 55 transcripts were identified from 15 genes involved in the flavonoid biosynthesis pathway (Figure 7B). The pathway demonstrated that metabolites such as eriodictyol, luteolin, and delphinidin were significantly accumulated in FR, possibly explaining the higher flavonoid concentrations in FR (Figure 7A). Seven DEGs, including CHS, FLS, DFR, ANR, and BZ1, were up-regulated, and similarly, seven DEGs were down-regulated in FR, possibly due to the complex regulatory processes of secondary metabolites. PGT1 was the only gene that was down-regulated in FR, and its expression correlated with phlorizin accumulation (Figure 7A). The accumulation of distinct metabolites was mainly consistent with the upregulation of the majority of transcripts in FR relative to the other three tissues (Figure 7B). In addition, the transcript is denoted as, CL6880.Contig1 annotated as flavonol synthase, exhibited higher expression in ST when compared to other tissues, presumably correlating with flavonol biosynthesis in ST (Figure 7B). These findings demonstrate the existence of a complex regulatory network between the accumulation of secondary metabolites and the level of gene expression, and further research is needed to investigate and validate these key genes involved in phenylpropanoid and flavonoid biosynthesis in *Trapa bispinosa*.

## Discussion

### Transcriptome Analysis of *Trapa bispinosa*

The application of transcriptome technology to identify and mine genes and transcripts, quantitative gene expression, and other research fields has been extensive. For example, multitudinous genes involved in flavonoid biosynthesis in *Ginkgo biloba* (Wu et al., 2018) and *Dendrobium officinale* (Yuan et al., 2020) have been identified using RNA sequencing technology. Although the origin and domestication of *Trapa bispinosa* were recently reported (Lu et al., 2022), the genes involved in the phenylpropanoid and flavonoid biosynthesis in different *Trapa bispinosa* tissues remain unclear. In this study, four samples of different *Trapa bispinosa* tissues were subjected to RNA sequencing. The number of annotated unigenes identified exceeded those reported in previous transcriptome sequencing of *Trapa bispinosa* roots and leaves samples (Li J. et al., 2017). The alignment results revealed that 60,634 unigenes (82.81%) of *Trapa bispinosa* exhibited high homology with pomegranate genes. Differential expression analysis revealed that the number of DEGs in FR-vs.-LF, FR-vs.-ST, and FR-vs.-RT were greater than 9,000. In addition, KEGG pathway analysis revealed that DEGs were predominantly distributed in secondary metabolite-related pathways, indicating that *Trapa bispinosa* FR may have exhibited robust metabolic activities.

A total of 805 candidate genes involved in phenylpropanoid biosynthesis and 225 candidate genes involved in flavonoid biosynthesis were identified. In addition, the expression of 76 (9.44%) phenylpropanoid biosynthesis-related genes and 27 (12.00%) flavonoid biosynthesis-related genes was higher in FR than in the other three tissues. In addition, the FR tissue of *Trapa bispinosa* had a higher phenylpropanoid and flavonoid biosynthesis activity, which is consistent with the results of the KEGG pathway analysis for DEGs. Multiple genes regulate the phenylpropanoid and flavonoid biosynthesis pathways, and these pathways play important roles in plant development and plant-environment interactions (Dong and Lin, 2021). Previous studies have demonstrated that multiple TFs regulate phenylpropanoid and flavonoid biosynthesis in plants. MYB (Ma et al., 2018; Tang et al., 2021), bHLH (Zhao et al., 2019), and WD40 (Mol et al., 2021), ate the most important TFs, while NAC (Yang et al., 2019), WRKY (Joshi et al., 2022), and bZIP (An et al., 2018) are the least important. In this study, 27 transcription factors that may play a role in the biosynthesis of phenylpropane or flavonoids were identified. These findings reveal the regulatory mechanism of phenylpropane or flavonoid biosynthesis in *Trapa bispinosa*.

### Metabolome Analysis of *Trapa bispinosa*

The shell of *Trapa bispinosa* has been utilized as a herbal medicine in China. Due to its phenolic and flavonoid compounds, it is also used as a pesticide (Adkar et al., 2014; Li F. et al., 2017). Currently, bioactive substances such as phenols and terpenes have been isolated from the shell of *Trapa bispinosa* (Adkar et al., 2014; Zhu, 2016; Xia et al., 2017), however, there are still numerous unidentified metabolites. In recent years, metabolome analysis technology has been widely used to detect and evaluate the changes in plant metabolites throughout growth and developmental stages (Yan N. et al., 2020; Zou et al., 2021). In this study, the metabolites of two different tissues of *Trapa bispinosa* were determined and identified using the targeted UPLC–MS/MS metabolomics approach. A total of 793 metabolites were identified, 167 of which were phenylpropane and flavonoids. The results of metabolite differential analysis demonstrated that the shell of *Trapa bispinosa* exhibited high metabolic activity of phenylpropane and flavonoid compounds, corroborating the results of transcriptome analysis. Altogether, these findings provide a theoretical basis for future use and development of the *Trapa bispinosa* shell.

### Integrated Analysis of the Transcriptome and Metabolome

It is difficult to determine the accumulation of metabolites solely by analyzing transcriptome expression levels because gene expression is regulated by numerous factors. Metabolites are the end products of biological processes, and levels of metabolites are regulated by numerous endogenous and exogenous factors (Yan N. et al., 2020; Urrutia et al., 2021). Consequently, integrating transcriptomics and metabolomics can better reveal the key functional genes involved in the regulation of metabolic pathways or molecular mechanisms of interest (Zhang et al., 2019; Yang et al., 2020; Sun et al., 2021). In this study, a comprehensive analysis of the transcriptome and metabolome indicated that numerous DEGs and DAMs participated in the same phenylpropanoid and flavonoid biosynthetic pathways, indicating that intense phenylpropanoid and flavonoid metabolic activity were exhibited in *Trapa bispinosa*. A total of 1,050 DEGs were significantly correlated with 62 DAMs in *Trapa bispinosa*, and one NAC family gene, unigene17,426 was found to be significantly positively correlated with seven flavonoids, suggesting that NAC genes may regulate flavonoids levels in *Trapa bispinosa*. *Trapa bispinosa* has complex regulatory mechanisms for metabolite and gene expression levels, as revealed by an analysis of the flavonoid biosynthesis pathway. To further explore the reasons for the differences in genes and metabolites associated with the biosynthesis pathways of phenylpropanoid and flavonoid, it is essential to investigate the correlations between genes and metabolites at different developmental stages of *Trapa bispinosa*. In general, these preliminary results demonstrate the correlation of metabolites and genes between the FR and LF and establish the foundation for future research on the specific mechanisms regulating the synthesis of secondary metabolites in *Trapa bispinosa*.

## Data Availability Statement

The datasets presented in this study can be found in online repositories. The names of the repository/repositories and accession number(s) can be found below: Bioproject accession number: PRJNA831559 and SRA accession number: SRR18907527-SRR18907538.

## Author Contributions

L-MW, H-XW, and TM contributed to the conception and design of the study. D-JY, S-JY, and Q-YC performed the statistical analysis. D-JY wrote the first draft of the manuscript. D-JY, S-JY, X-YS, and Q-YC wrote sections of the manuscript. All authors contributed to manuscript revision, read, and approved the submitted version.

## Conflict of Interest

The authors declare that the research was conducted in the absence of any commercial or financial relationships that could be construed as a potential conflict of interest.

## Publisher’s Note

All claims expressed in this article are solely those of the authors and do not necessarily represent those of their affiliated organizations, or those of the publisher, the editors and the reviewers. Any product that may be evaluated in this article, or claim that may be made by its manufacturer, is not guaranteed or endorsed by the publisher.

## Footnotes

1
http://www.metaboanalyst.ca/
