# Supplementary figures and images for: Integrative Analysis of the Transcriptome and Metabolome Reveals Genes Involved in Phenylpropanoid and Flavonoid Biosynthesis in the Trapa bispinosa Roxb
Source: Front Plant Sci. 2022 Jul 7;13:913265. doi: 10.3389/fpls.2022.913265 (PMC9302371; doi:10.3389/fpls.2022.913265)

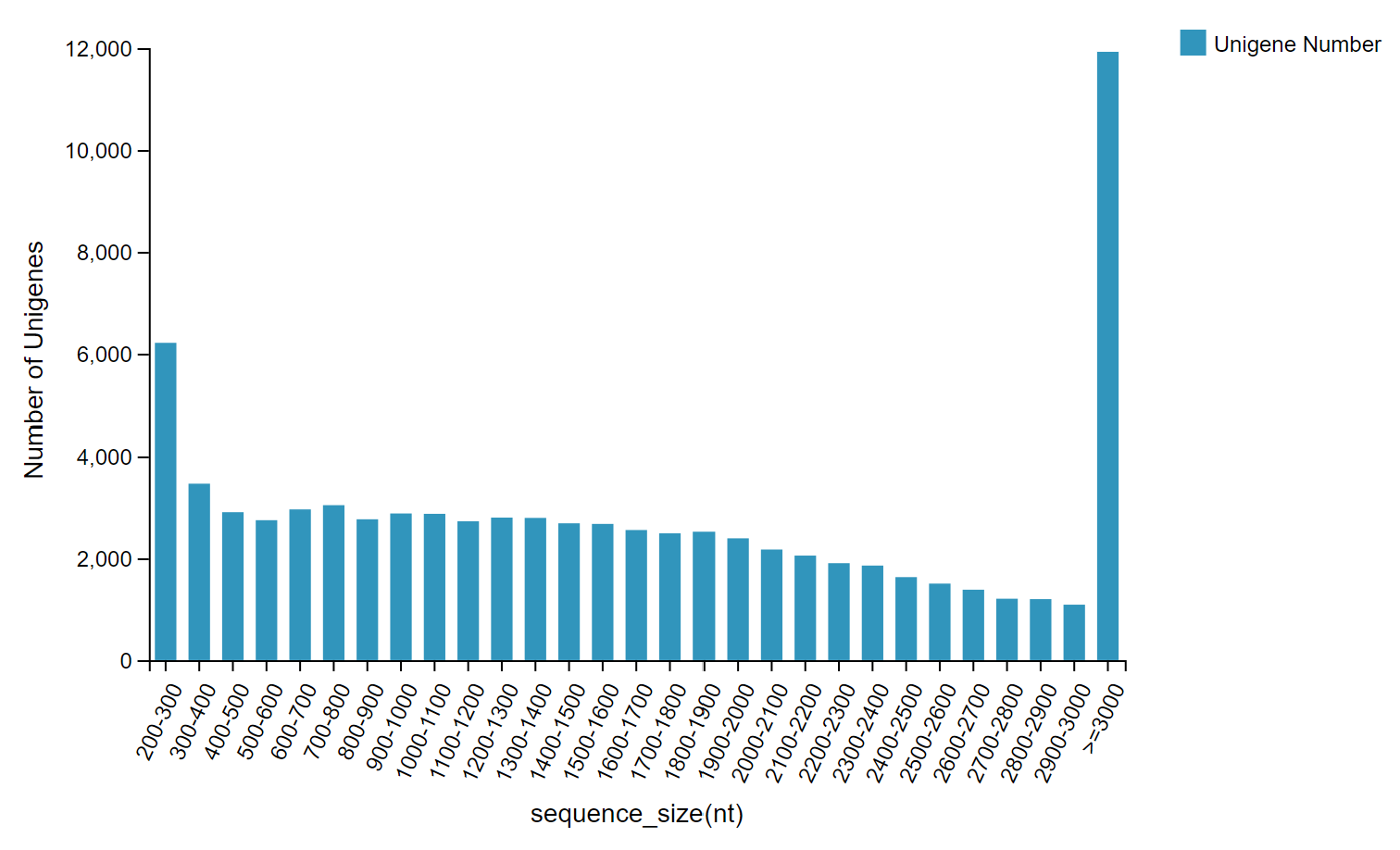

Supplement: Supplementary file 1 [file Data_Sheet_1.zip › Supplementary Materials/Supplementary Figure 1.png]

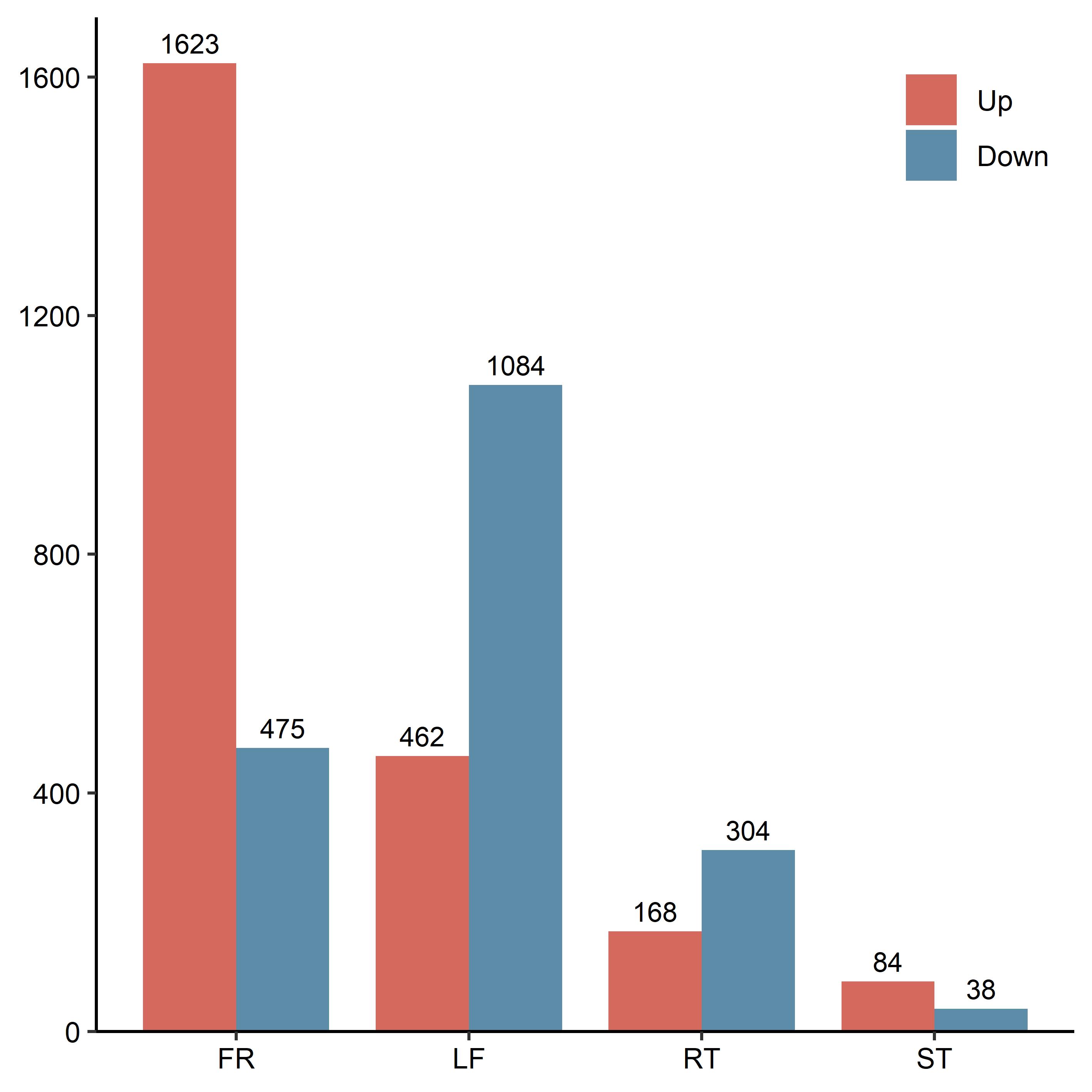

Supplement: Supplementary file 1 [file Data_Sheet_1.zip › Supplementary Materials/Supplementary Figure 12.jpg]

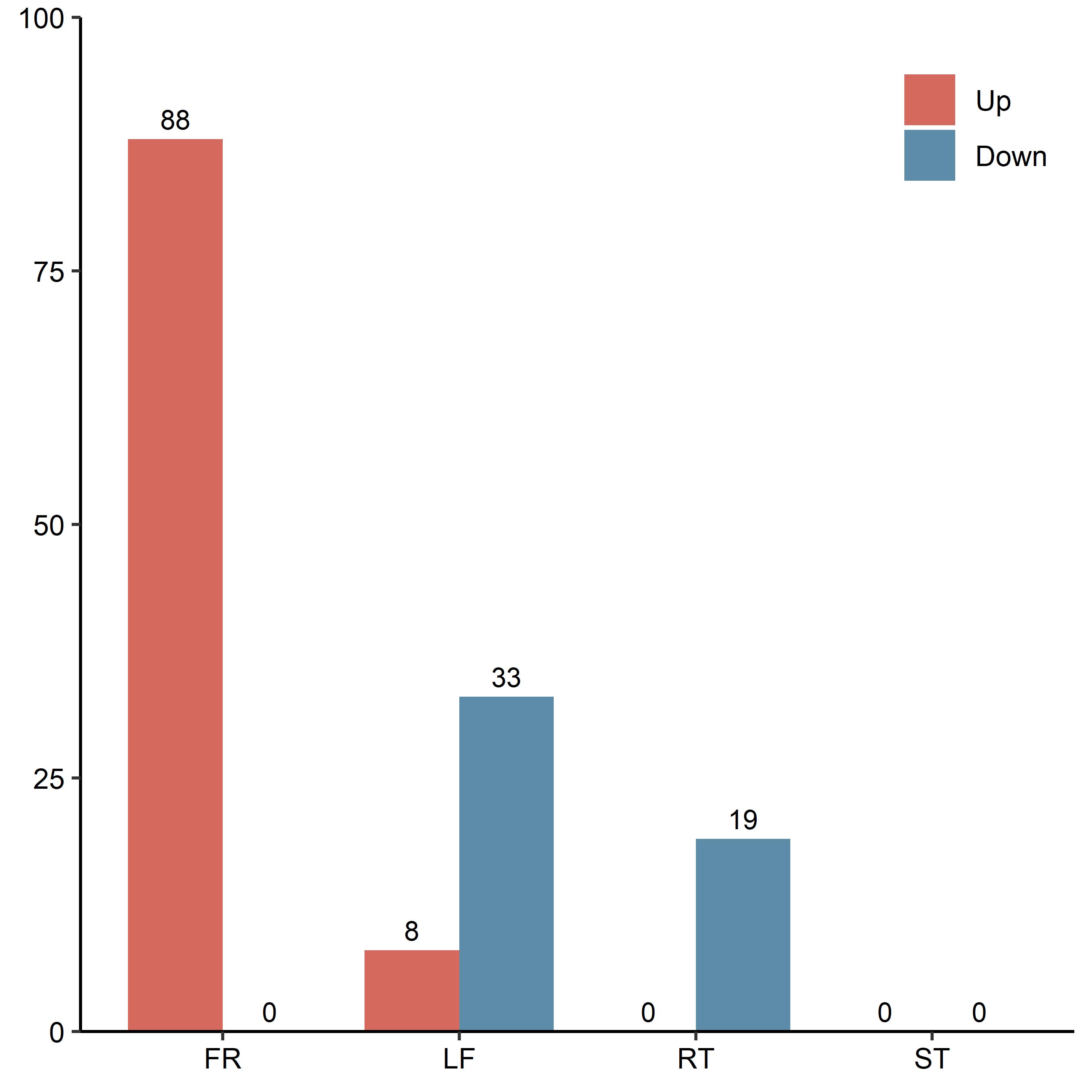

Supplement: Supplementary file 1 [file Data_Sheet_1.zip › Supplementary Materials/Supplementary Figure 13.jpg]

TIC CURVE OF QC SAMPLES

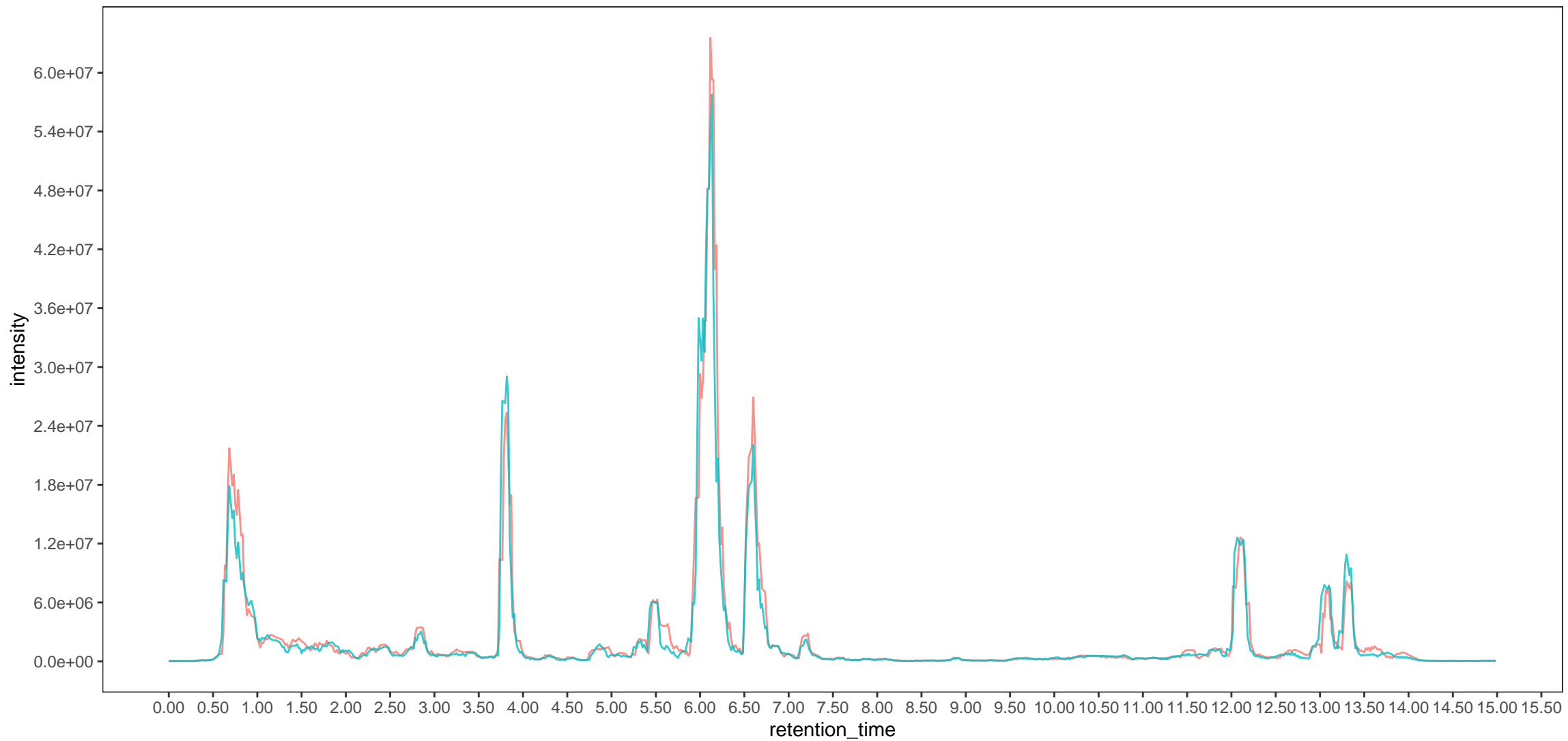

Supplement: Supplementary file 1 [file Data_Sheet_1.zip › Supplementary Materials/Supplementary Figure 14.pdf]

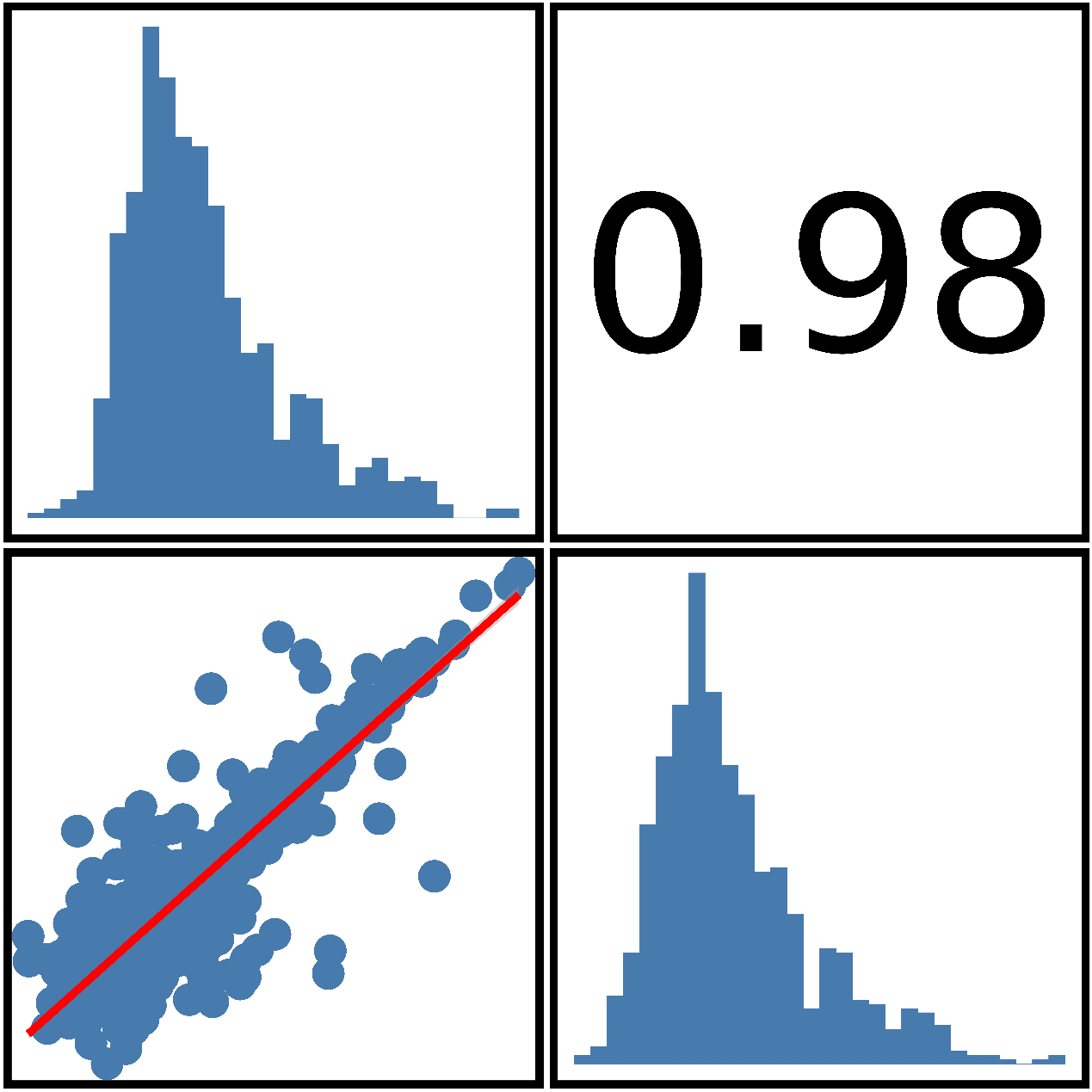

Supplement: Supplementary file 1 [file Data_Sheet_1.zip › Supplementary Materials/Supplementary Figure 15.png]

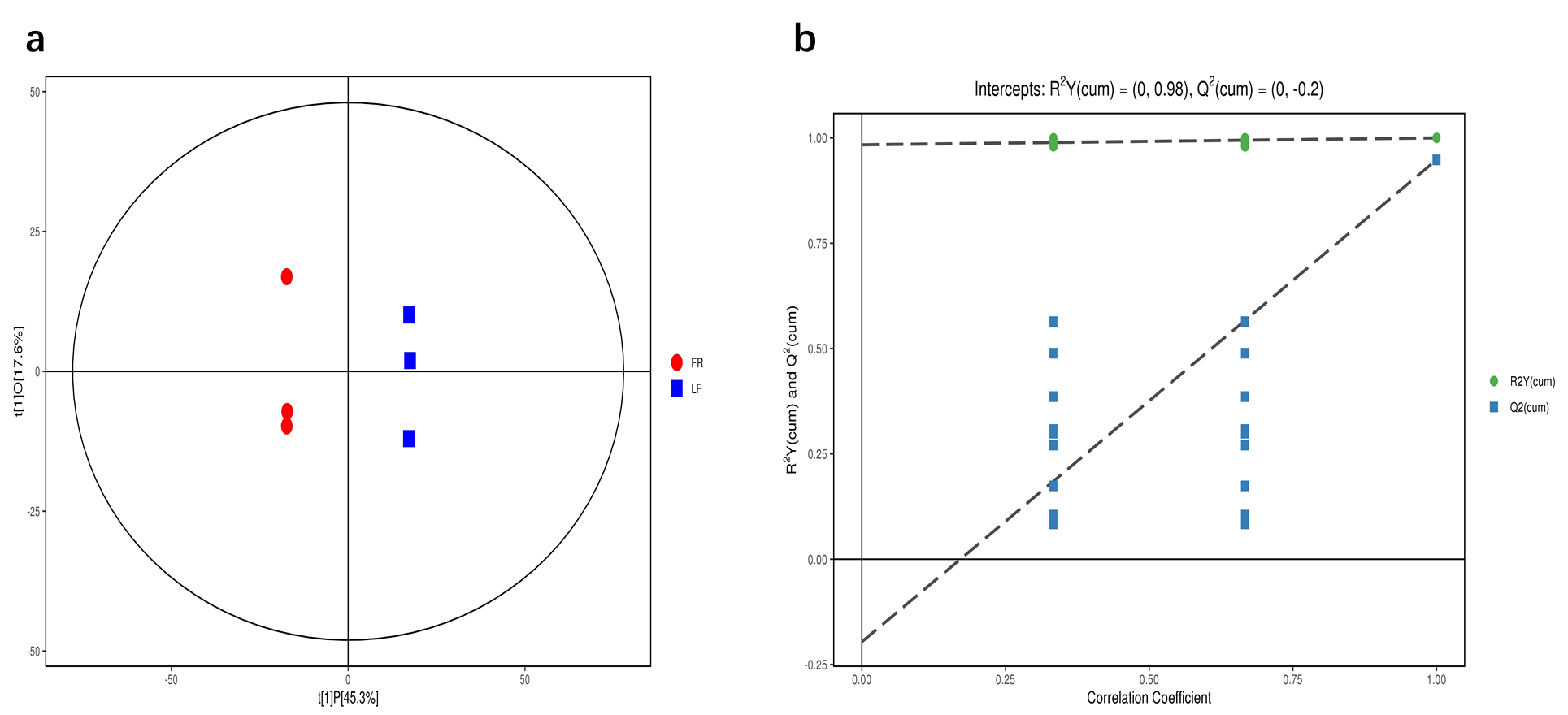

Supplement: Supplementary file 1 [file Data_Sheet_1.zip › Supplementary Materials/Supplementary Figure 16.png]

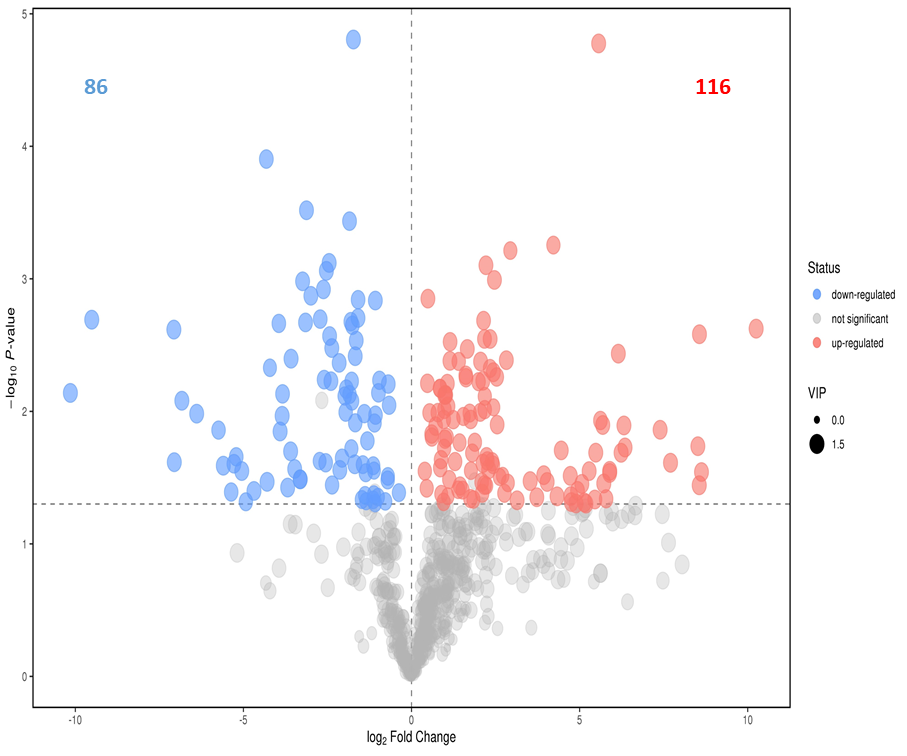

Supplement: Supplementary file 1 [file Data_Sheet_1.zip › Supplementary Materials/Supplementary Figure 17.png]

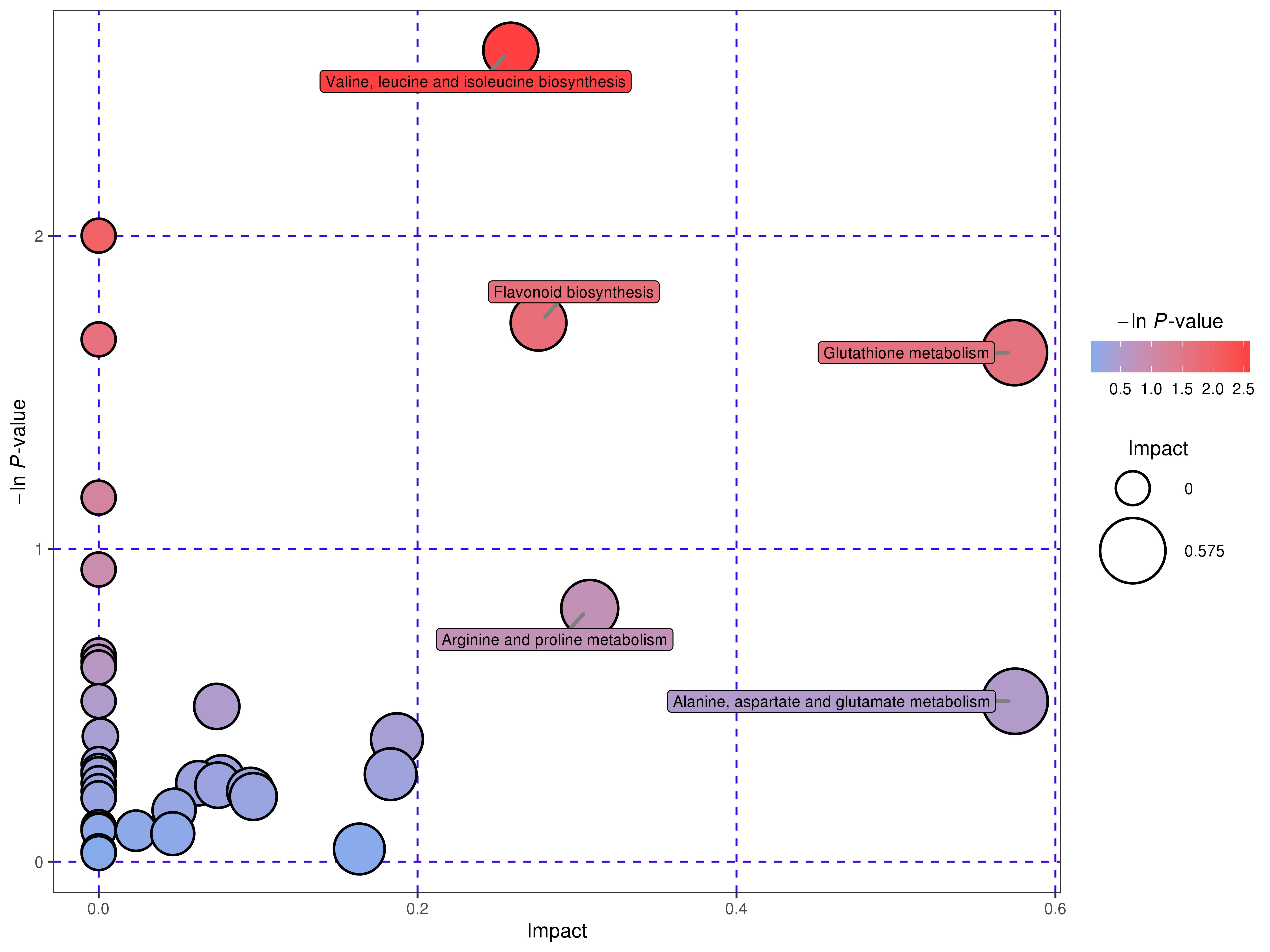

Supplement: Supplementary file 1 [file Data_Sheet_1.zip › Supplementary Materials/Supplementary Figure 18.jpg]

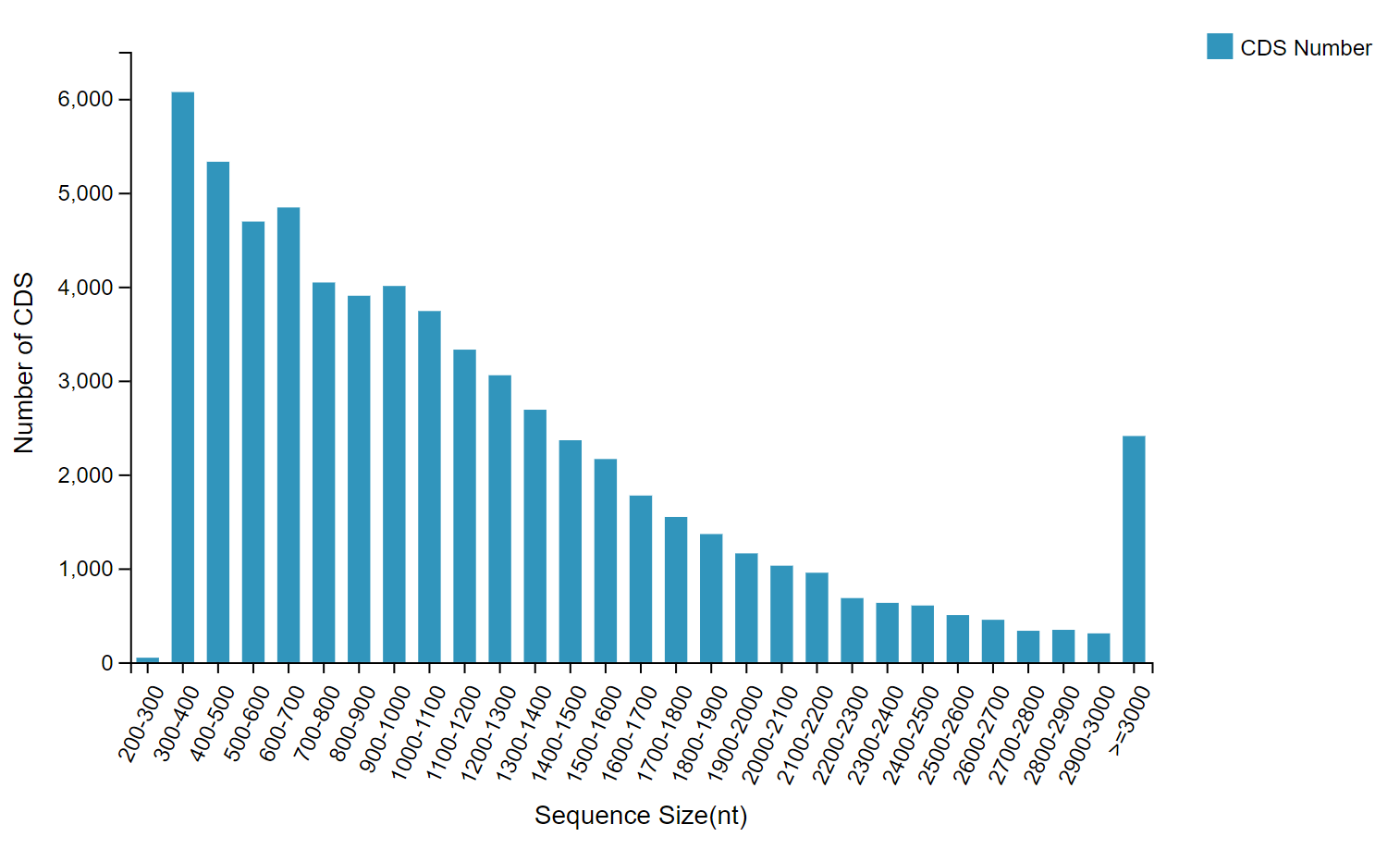

Supplement: Supplementary file 1 [file Data_Sheet_1.zip › Supplementary Materials/Supplementary Figure 2.png]

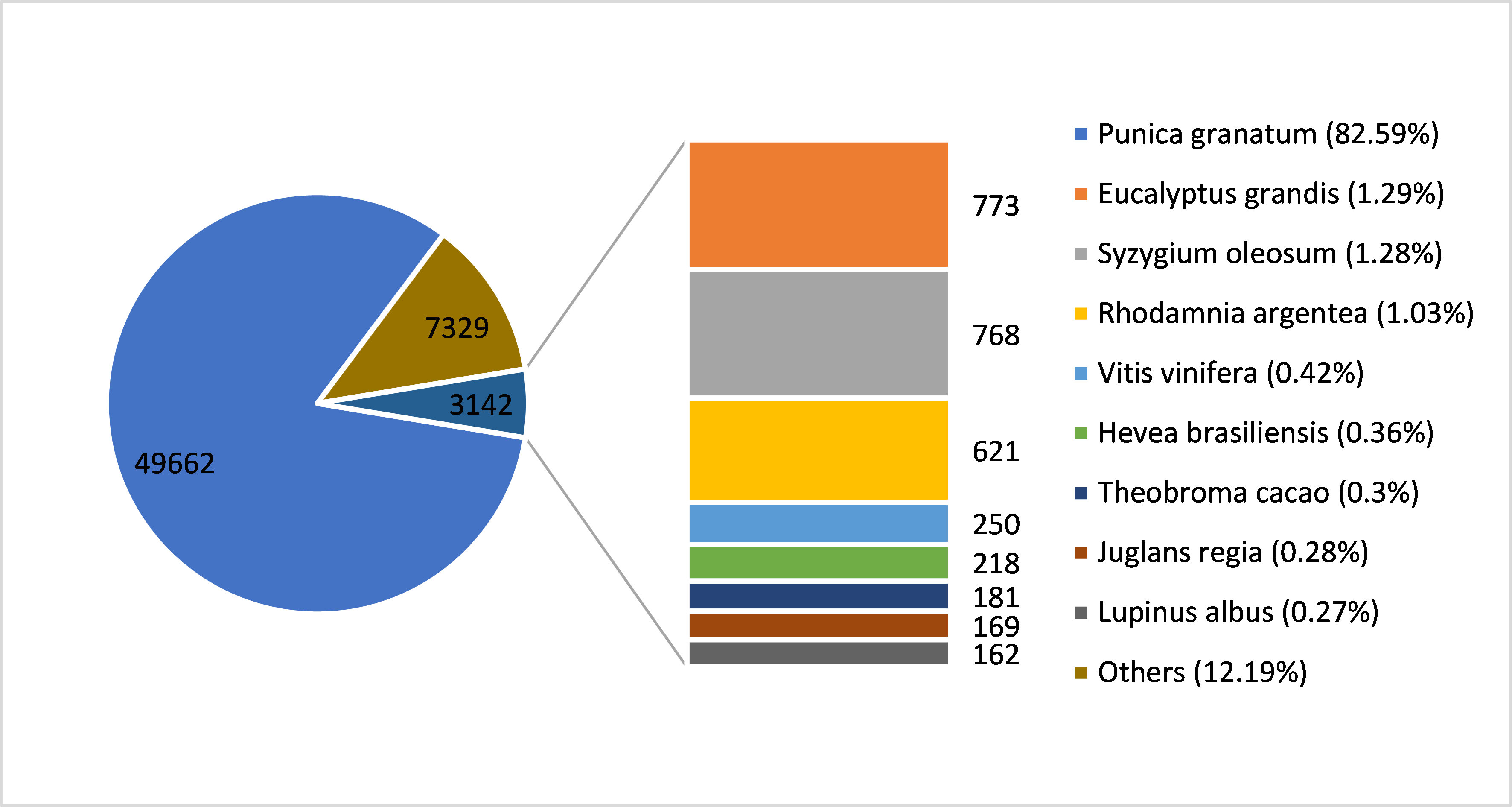

Supplement: Supplementary file 1 [file Data_Sheet_1.zip › Supplementary Materials/Supplementary Figure 3/Figure S3-1.png]

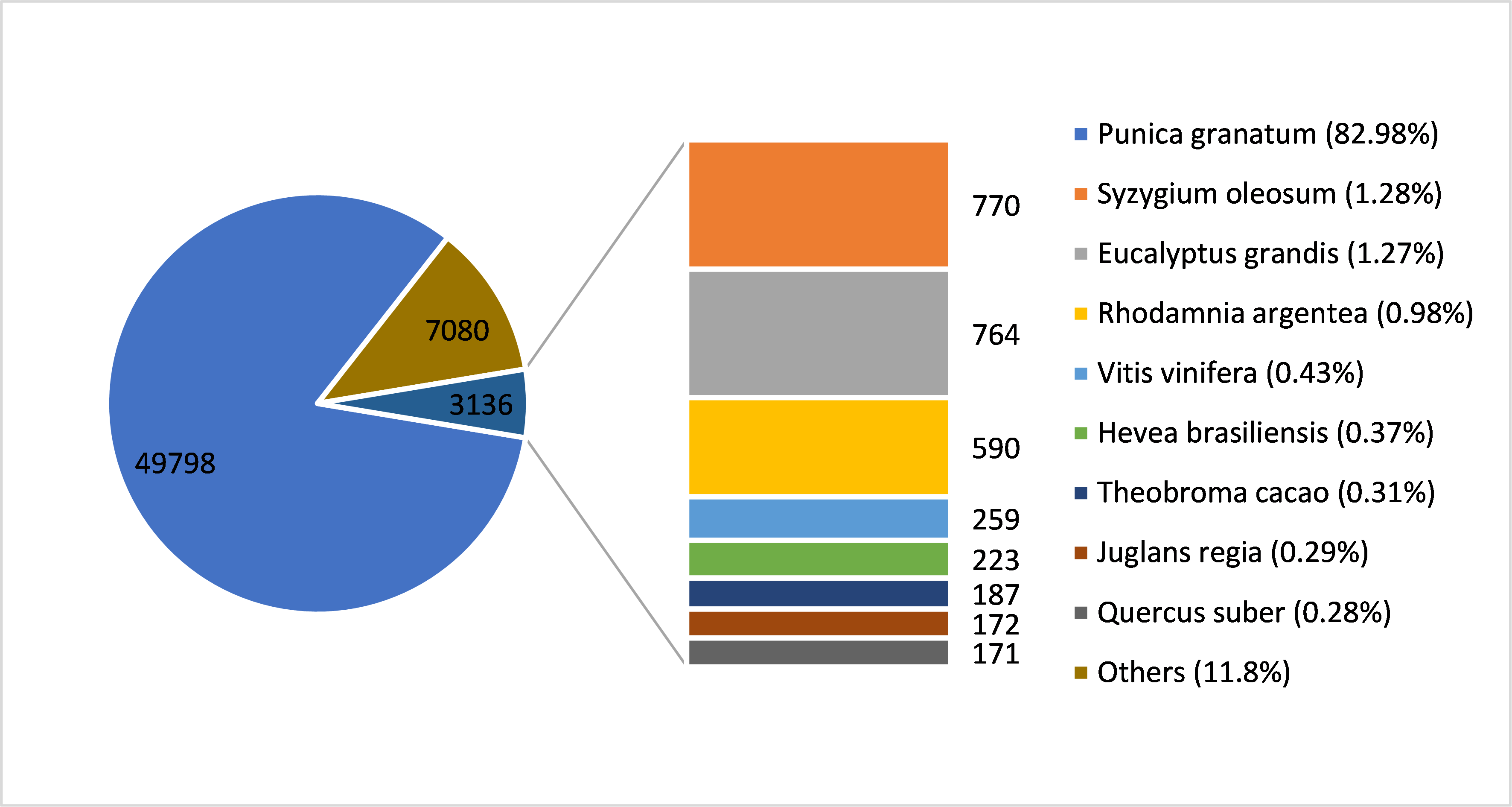

Supplement: Supplementary file 1 [file Data_Sheet_1.zip › Supplementary Materials/Supplementary Figure 3/Figure S3-2.png]

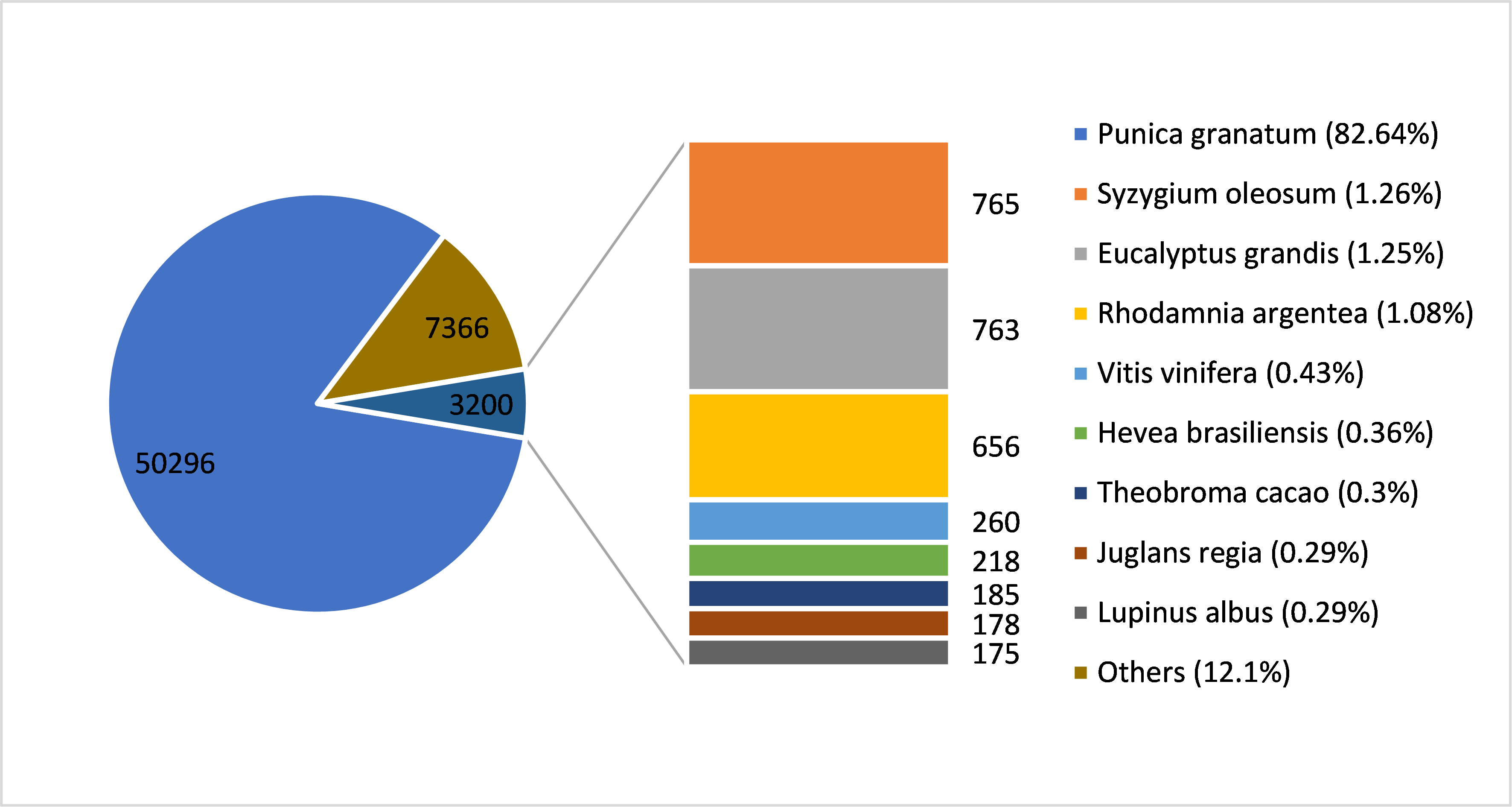

Supplement: Supplementary file 1 [file Data_Sheet_1.zip › Supplementary Materials/Supplementary Figure 3/Figure S3-3.png]

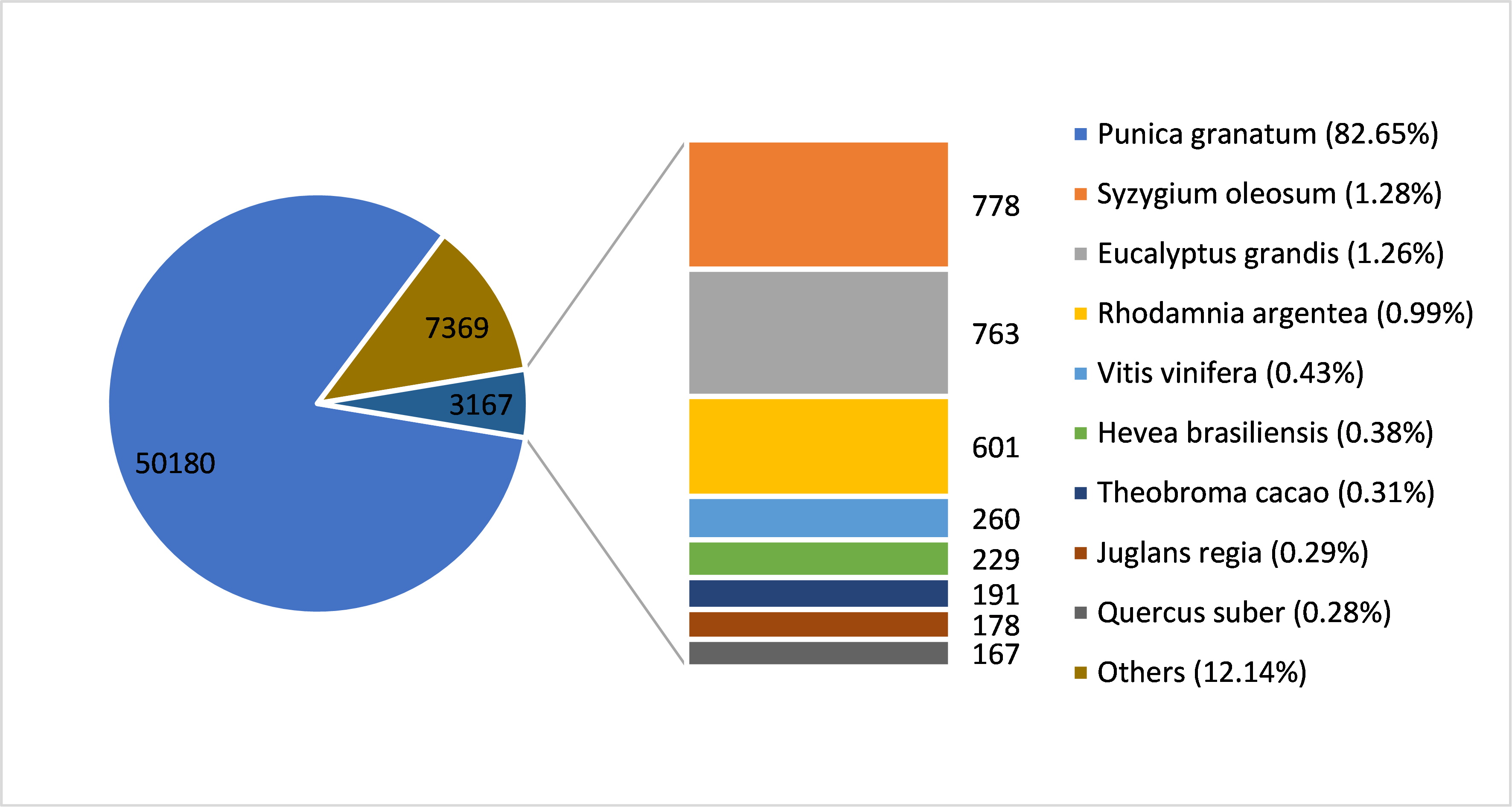

Supplement: Supplementary file 1 [file Data_Sheet_1.zip › Supplementary Materials/Supplementary Figure 3/Figure S3-4.png]

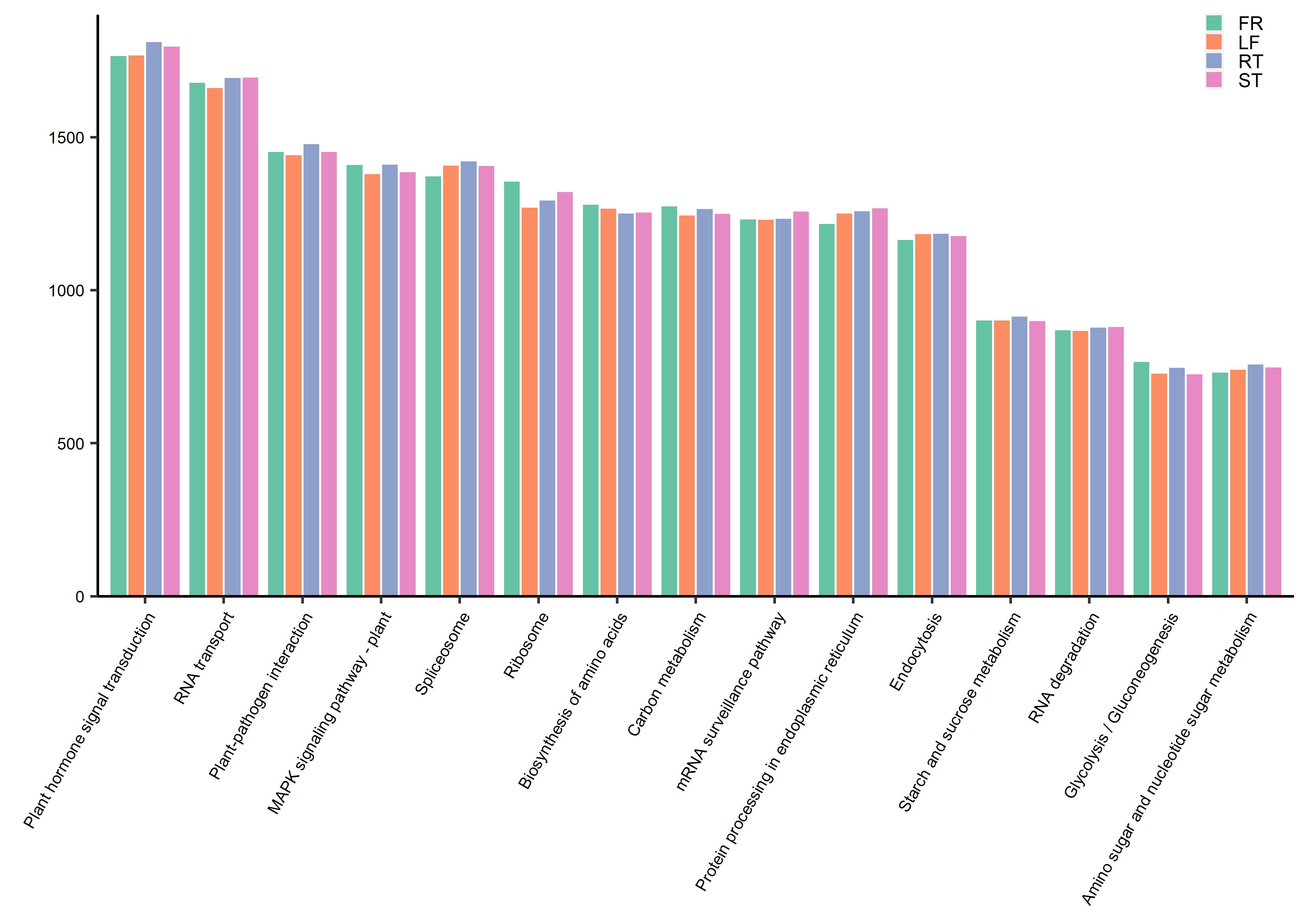

Supplement: Supplementary file 1 [file Data_Sheet_1.zip › Supplementary Materials/Supplementary Figure 5.jpg]

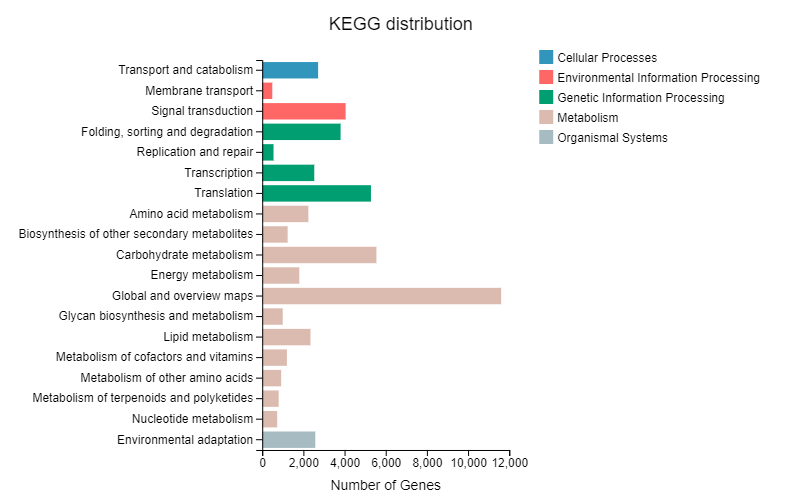

Supplement: Supplementary file 1 [file Data_Sheet_1.zip › Supplementary Materials/Supplementary Figure 6.png]

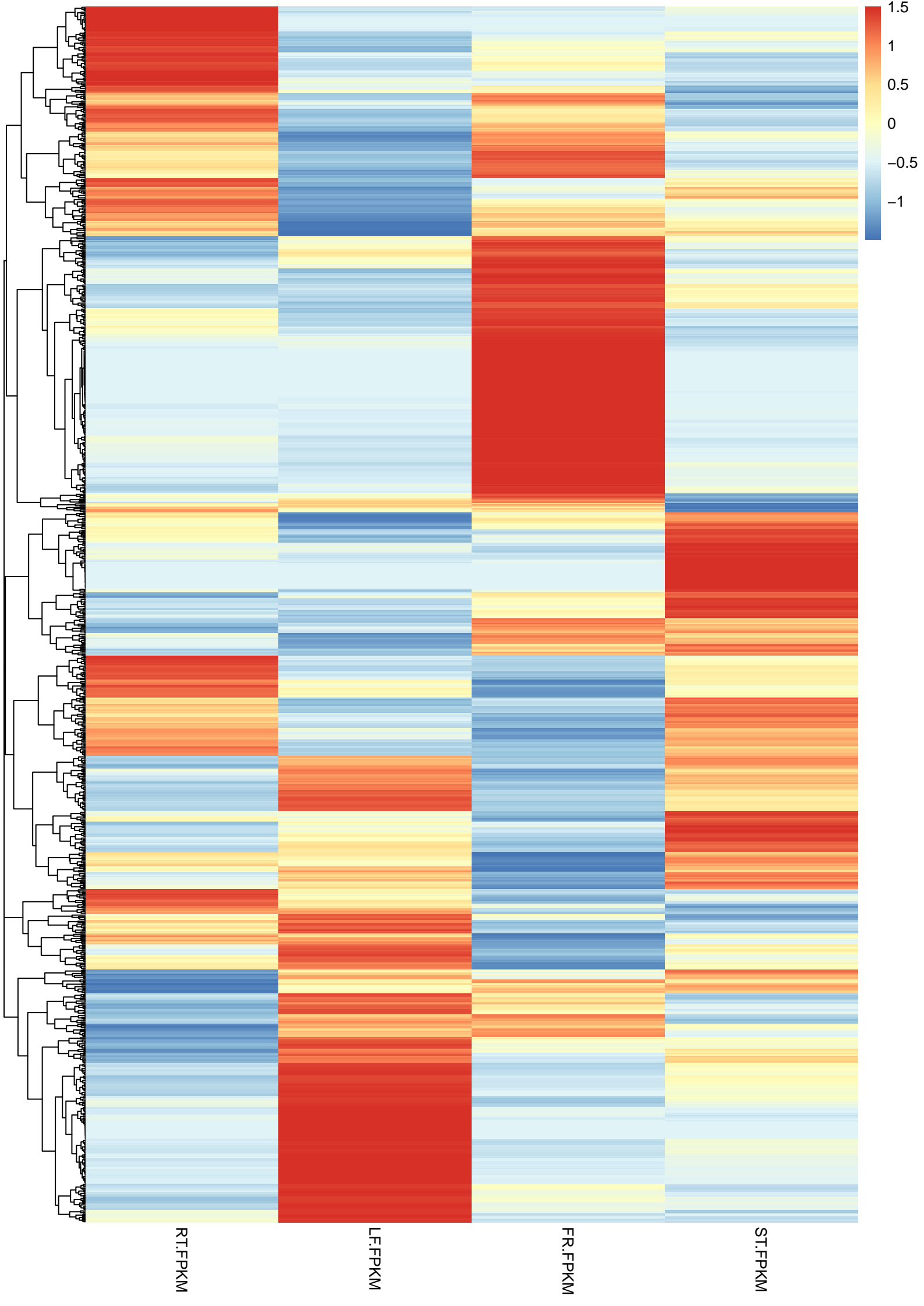

Supplement: Supplementary file 1 [file Data_Sheet_1.zip › Supplementary Materials/Supplementary Figure 7.pdf]

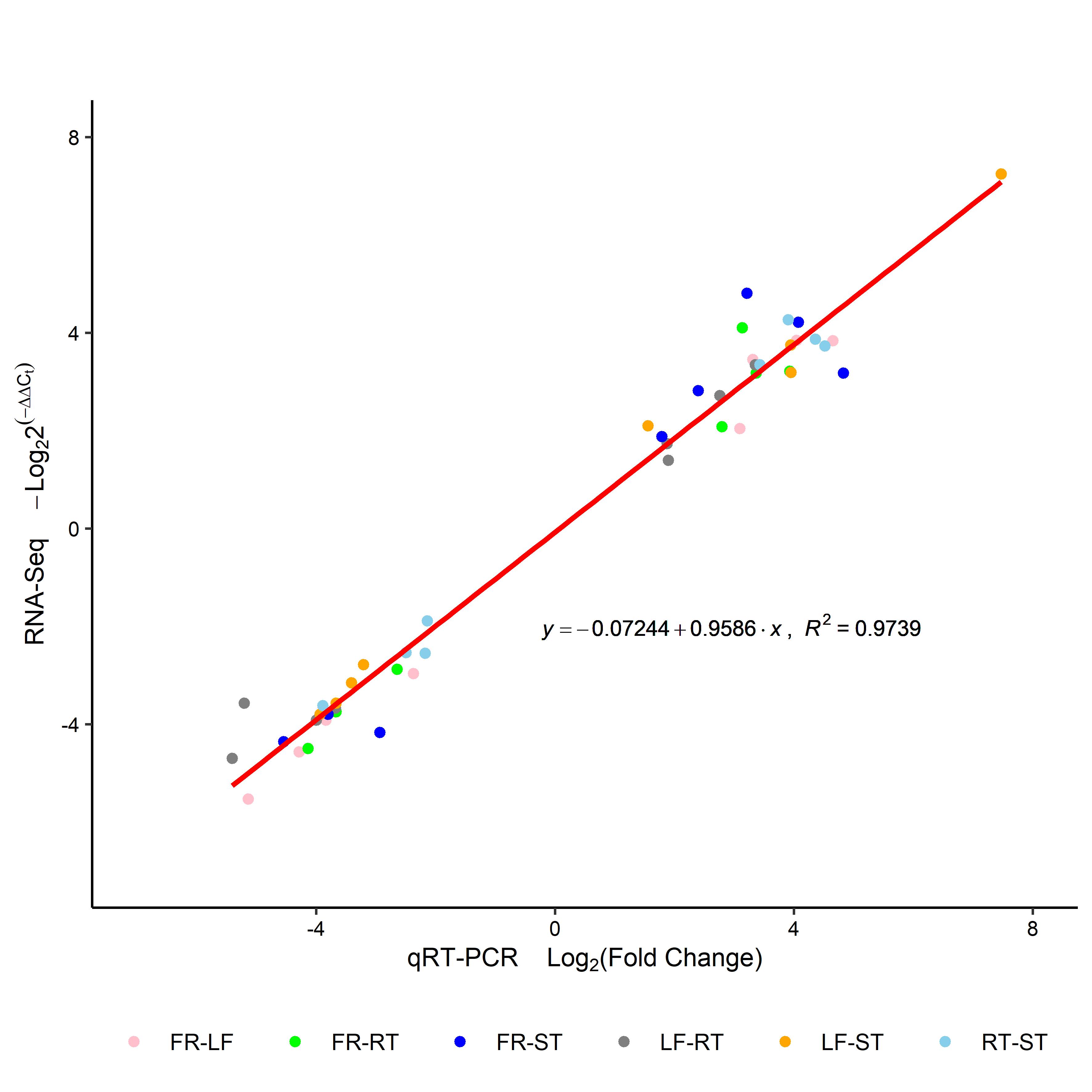

Supplement: Supplementary file 1 [file Data_Sheet_1.zip › Supplementary Materials/Supplementary Figure 8.jpg]

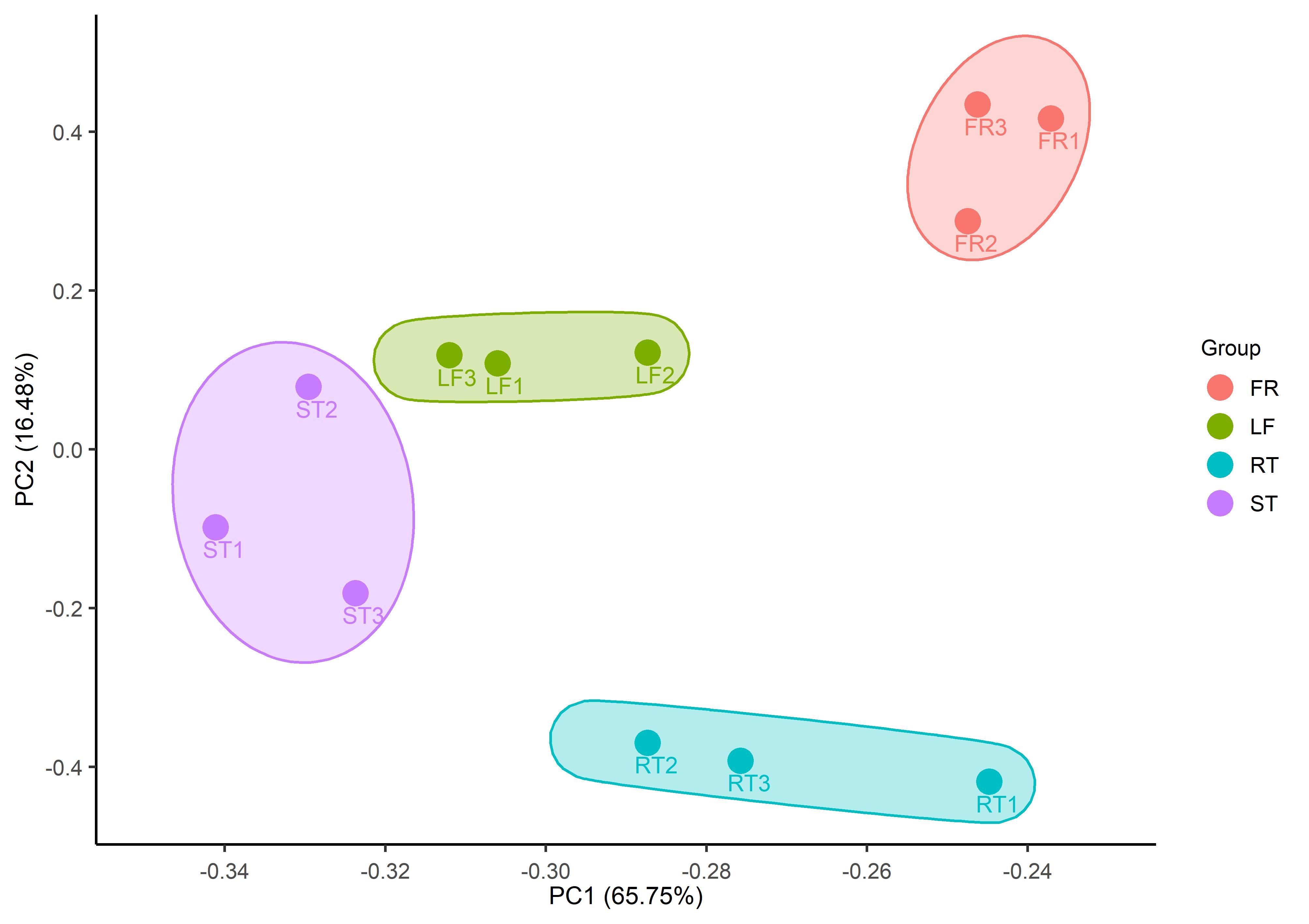

Supplement: Supplementary file 1 [file Data_Sheet_1.zip › Supplementary Materials/Supplementary Figure 9.jpg]

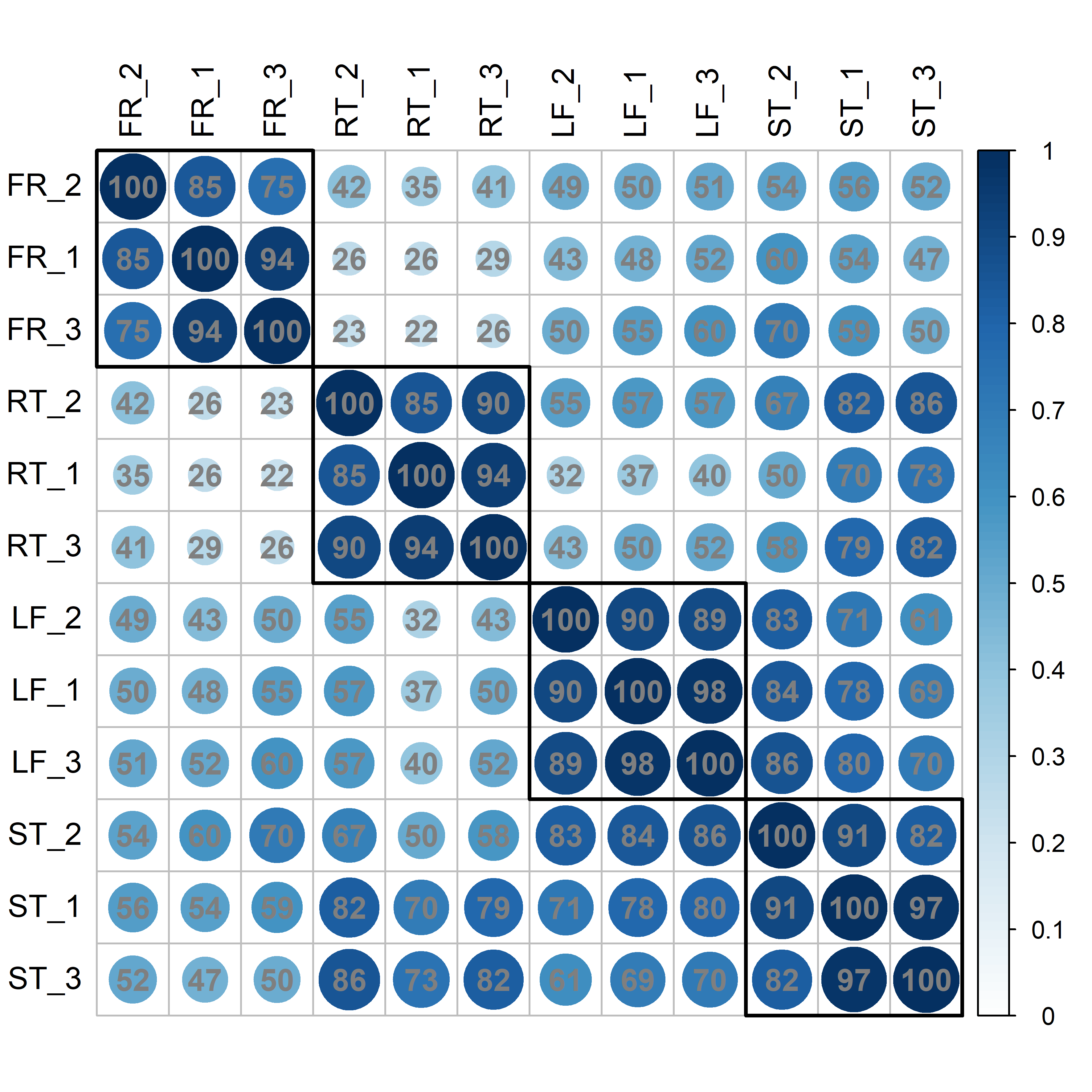

Supplement: Supplementary file 1 [file Data_Sheet_1.zip › Supplementary Materials/Supplementary Figure 10.png]

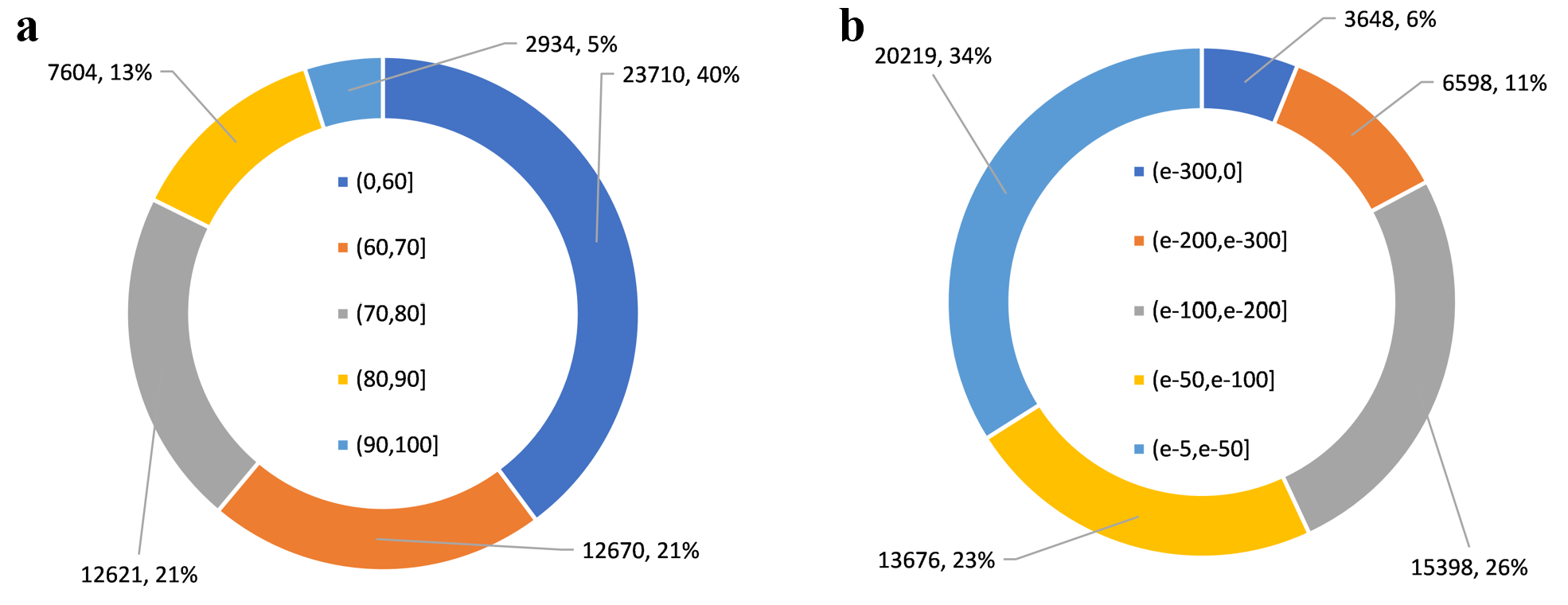

Supplement: Supplementary file 1 [file Data_Sheet_1.zip › Supplementary Materials/Supplementary Figure 4.png]
